# Inventory of Medicinal Plants Used Traditionally to Manage Kidney Diseases in North-Eastern Morocco: Ethnobotanical Fieldwork and Pharmacological Evidence

**DOI:** 10.3390/plants10091966

**Published:** 2021-09-20

**Authors:** Noureddine Bencheikh, Amine Elbouzidi, Loubna Kharchoufa, Hayat Ouassou, Ilyass Alami Merrouni, Hamza Mechchate, Imane Es-safi, Christophe Hano, Mohamed Addi, Mohamed Bouhrim, Bruno Eto, Mostafa Elachouri

**Affiliations:** 1 Laboratory of Bioresources, Biotechnology, Ethnopharmacology and Health, Faculty of Sciences, Mohammed First University, Oujda 60040, Morocco; bencheikh_noureddine1718@ump.ac.ma (N.B.); amine.elbouzidi@ump.ac.ma (A.E.); l.kharchoufa@ump.ac.ma (L.K.); hayatouassou@gmail.com (H.O.); alami.ilyass.90@gmail.com (I.A.M.); mohamed.bouhrim@gmail.com (M.B.); elachouri.mostafa@ump.ac.ma (M.E.); 2 Laboratory of Biotechnology, Environment, Agrifood and Health, University of Sidi Mohamed Ben Abdellah, P.O. Box 1796, Fez 30000, Morocco; Hamza.mechchate@usmba.ac.ma; 3 Laboratoire de Biologie des Ligneux et des Grandes Cultures, INRA USC1328, Orleans University, CEDEX 2, 45067 Orléans, France; christophe.hano@univ-orleans.fr; 4 Laboratoire d’Amélioration des Productions Agricoles, Biotechnologie et Environnement (LAPABE), Faculté des Sciences, Université Mohammed Premier, Oujda 60040, Morocco; m.addi@ump.ac.ma; 5 Laboratoire TBC, Laboratory of Pharmacology, Pharmacokinetics, and Clinical Pharmacy, Faculty of Pharmaceutical and Biological Sciences, University of Lille, 3, rue du Professeur Laguesse, B.P. 83, F-59000 Lille, France; eto.brunoalves17@gmail.com

**Keywords:** Ethnobotany, ethnopharmacology, traditional medicine, renal diseases, folk medicine, traditional knowledge, kidney problems, lithiasis, calculus, diuretic

## Abstract

Kidney disease is one of the most common health problems and kidney failure can be fatal. It is one of the health disorders associated with extreme pain and discomfort in patients. In developing countries, such as Morocco where socioeconomic and sanitary conditions are precarious, medicinal plants are considered the primary source of medication. In the present work an ethnobotanical survey was conducted in a remote area of North-Eastern Morocco and we focused on (1) establishing a record of medicinal plants used traditionally by local people to treat kidney diseases and (2) correlate the obtained ethnomedical use with well-studied pharmacological evidence. From February 2018 to January2020, information was gathered from 488 informants using semi-structured questionnaires. The data were analyzed using three quantitative indices: The use value (UV), family use value (FUV), and informant consensus factor (ICF). A total of 121 plant species belonging to 57 botanical families were identified to treat kidney diseases. The families most represented were Asteraceae (14 species), followed by Lamiaceae (12 species) and Apiaceae (10 species). The most commonly used plant parts were leaves, followed by the whole plant and they were most commonly prepared by decoction and infusion. The highest value of the (UV) index was attributed to *Herniaria hirsuta* L. (UV = 0.16), and the highest family use value (FUV) was assigned to Caryophyllaceae with (FUV = 0.163). Regarding the informant consensus factor (ICF), this index’s highest values were recorded for kidney stones (ICF = 0.72). The use of 45% of the selected plants were validated based on literature review. This study helped document and preserve crucial traditional plant knowledge of 121 plant species used to treat kidney problems that can be used in the search for new biologically active compounds through more upcoming pharmacological studies.

## 1. Introduction

Nowadays, kidneys and their problems have gained increasing interest concomitant with life changes, industrialization and malnutrition. Plants have always played a significant role in traditional medicine in underdeveloped countries and have also been an integral part of local communities’ history and cultural practices [1]. Medicinal plants have been recognized for centuries as a rich source of medicinal agents for preventing and treating a variety of ailments in Morocco [2]. Several researches conducted in different regions of Morocco indicated that people excessively use medicinal plants to meet their healthcare needs in Morocco (at least 75% of the population) [3,4] and it is due to several factors, such as the high cost of conventional medicines, the lack of adequate sanitary facilities, and frangible socioeconomic conditions of users, especially those living in poor, remote areas and also their safety and low incidence of adverse effects [5].

As other regions of Morocco, people living in North-Eastern Morocco have a common cultural past that dates back to the Arab civilization in the seventh century. The original cumulative culture has maintained a well-developed traditional knowledge of medicinal plants’ uses that form the basis of the traditional medical system existing until now [6,7]. Unfortunately, this local cultural and natural heritage is threatened with extinction. The decrease of these phyto-therapeutic practices and the degradation of phyto-genetic resources are due to several factors, mainly the lack of documentary databases related to traditional medical practices and the scarcity of ethnobotanical information archives aggravate this natural and cultural heritage loss.

Regarding, these ancestral medical practices in this country, we found that there are many investigations carried out in different regions of Morocco that deal with traditional use of medicinal plants for the treatment of renal diseases. In fact, an ethnobotanical survey conducted in the Fez-Meknes region was able to document traditional knowledge related to the 69 plant species belonging to 38 families, used as traditional remedies for the treatment of kidney diseases in this region [8]. In the Boulemane region of Morocco, Jouad et al. (2001) conducted an ethnobotanical survey to document traditional medicinal practices related to medicinal plants used for the treatment of diabetes and kidney disease, among which they identify 33 medicinal plants used specifically to treat kidney problems [9]. In addition, a study led by Khouchlaa et al. (2016) in the Rabat region provided a catalogue of 35 medicinal plants with information on therapeutic practices for treating urinary lithiasis [10]. All these ethnobotanical fieldworks cited are practically concentrated on a part of the Moroccan territory, which shows that the ancestral medical practices in this country have remained to be preserved with regard to kidney diseases.

To the best of our knowledge, no ethnobotanical survey on the use of medicinal plants used in treating kidney diseases has been conducted in North-Eastern Morocco, appealing this study to be conducted with the objective of (1) record, evaluate, and document medicinal plants and know-how related used by local people, in the control and healing of renal disorders, in six provinces of the North-Eastern region of Morocco, (2) make a quantitative analysis of traditional knowledge assigned to species inventoried, (3) provide pharmacological and toxicological data of the plant species listed in the present paper.

## 2. Results and Discussion

### 2.1. Socio-Demographic Data

#### 2.1.1. Global Data

In Table 1, we regrouped the information on the participants’ sociodemographic characteristics in this study. The variable including age, gender, education level, income, and attitude toward medication. These data showed that 488 local informants were interviewed, including 476 non-specialists and 12 health herbalists (care professionals). The use of medicinal plants in the area of study is widespread in all age groups. As indicated in Table 1, participants in the age group [46–65 years] have more knowledge of medicinal plants than other age groups, with a frequency of use 53%, followed by the group [25–45 years] with 27%, age group [over 65 years] with 11% and the last group [under 25 years], with a percentage 9%. These results confirm the data indicated previously in other ethnobotanical studies conducted in other areas in Morocco [11,12]. The high proportion of participants was dominated by women, with 58% followed by men with 42%. The high possession of the traditional phytotherapy knowledge, detained by women, could be explained by the nature of women’s behaviors within their families; in fact, the women were frequently sitting at home and are responsible for the care of their children and to maintain the health of their families in the most effective and economic ways [13]. So, we can say that women were more connected to traditional practices than men. These results are consistent with other national work results [6,14,15,16]. Regarding the level of education, the results showed that 59% of the respondents were illiterate, followed by secondary and primary education categories, with percentages, respectively, of 17% and 14%. However, people with a university-level education represented a low percentage of 9%. These observations showed that traditional remedies used by people living in this region of study to treat renal diseases are affected by the educational level and age of participants. Our findings resonate with other results observed in other ethnobotanical fieldworks conducted in other Morocco regions [17,18,19].

#### 2.1.2. Attitude of the Population toward Pattern of Uses

In this part of the text and for convenience, we divided medical practices, adopted by the population in North-Eastern Morocco, for treating renal diseases into three categories: those using only medicinal plants for these purposes, those using conventional medicine, and those using both traditional and conventional medicine. As indicated in Table 1, the population’s attitude in this region toward the treatment of renal diseases is variable. These data highlight the great diversity in patterns of use. The majority of interviewers cited traditional healthcare as their first-choice treatment option when they felt sick, with a percentage equaling to 54%, followed by the second choice, corresponding to the use of both conventional and herbal medicine, with a percentage of 33%, and in the third choice relative to the persons using exclusively modern medicine, with a percentage of 13%. Within the context of a dual health care system (traditional and western), the most significant determinants behind the participants’ attitude towards traditional medicine were the socio-economic factors and the residence of the users. Several factors were behind the driving force leading the majority of the interviewers toward traditional medicine. The results regrouped in Table 1 and Table 2 showed that the total people interviewed were living in remote areas and had a low socio-economic level. In addition to the lacking money and the high cost of modern medical treatment of renal diseases, the travel to cities, where the patients could have access to health facilities, constitutes a barrier to reach modern medicine and pull factors that attract people into seeking traditional treatments in the local area of study. This is congruent with studies conducted among populations in other Morocco regions [20].

Based on the information mentioned above, we deduced that informal health care approaches “traditional medication” have been reported to be shared among people living in this region, especially for renal diseases. Despite the population in this region’s lack of trust in the modern healthcare system, our findings confirm that patients still consider traditional medical practices a better option than conventional healthcare approaches.

So, according to these observations, we can say that the socio-economic conditions, patients’ residence, culture, and tradition influenced the user’s decision to use traditional healthcare approaches.

#### 2.1.3. Source of Information

Among participants who chose informal healthcare as their first-choice treatment option, their subsequent decision to use standard healthcare options depended on their experiences or their initial interaction with the older and herbalists, when that exists. According to our results, most parts of ethnobotanical information generated from this inquiry were given by people living in remote areas. Based on this inquiry’s ethnobotanical information, we deduced that the accumulated experiences with age are the primary source of information at the local level. The highest age respondents provide more reliable information because they hold much of the oral tradition’s ancestral knowledge. However, the young generation detained less information related to traditional knowledge because they were influenced by modernization and exotic culture and the tendency to disinterest and the gradual mistrust of this herbal medicine. So, the present-day, the substantial holder of traditional knowledge, which is becoming very old, and the lack of interest among the younger generation as well as their tendency to migrate to cities to ensure their basic needs, could harm the transmission of the traditional know-how on medicinal plants of the elderly to the young people.

Consequently, the traditional indigenous knowledge that has been transferred orally, which is fast disappearing, is in danger, and there is a possibility of losing this wealth of knowledge shortly. Indeed, this traditional knowledge on phytotherapy, which is transmitted from one generation to the next, is on the verge of extinction if no effort is made to save it [21].

### 2.2. Diversity of Plants Species Used to Treat Kidney Diseases

In the present study, 121 species of medicinal plants belonging to 57 families were used to treat kidney diseases. Ethnobotanical information related to these plants’ use was documented, including vernacular names, traditional uses, parts used, method of preparation, and route of administration (Table 3).

**Figure 1:**
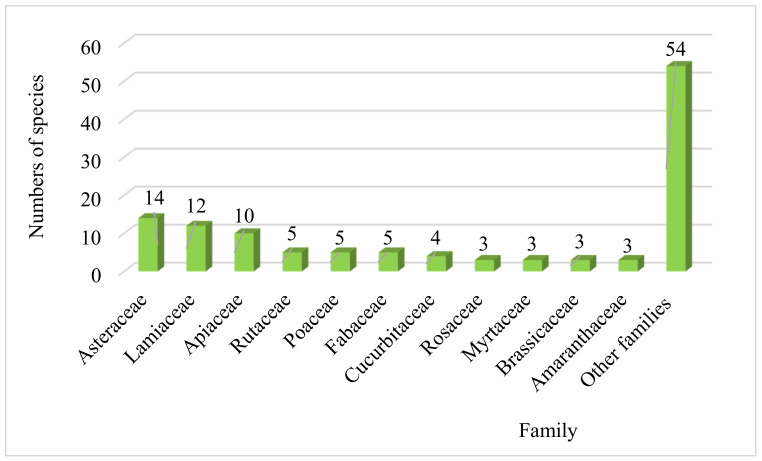
Dominant botanical families.

The dominated families that have been used to treat and relieve renal disorders were the Asteraceae (14 species), followed by the Lamiaceae (12 species), the Apiaceae (10 species), Rutaceae, Poaceae and Fabaceae (5 species) each, Cucurbitaceae with (4 species), Rosaceae, Myrtaceae, Brassicaceae and Amaranthaceae with (3 species for each), while the other families represent less than three species (Figure 1). The predominance of Asteraceae, Lamiaceae, and Apiaceae, has already been proven in several ethnobotanical studies carried out in other Moroccan regions [14,22,23,24], as well as in other countries such as Turkey [25] and Italy [26]. Furthermore, the predominance of these plant families has already been confirmed in the results of specific ethnobotanical work for kidney disorders conducted in the Moroccan territory [17,22,27]. On the other hand, these botanical families dominate the Moroccan flora and are also almost omnipresent in the Moroccan territory [28].

**Figure 2:**
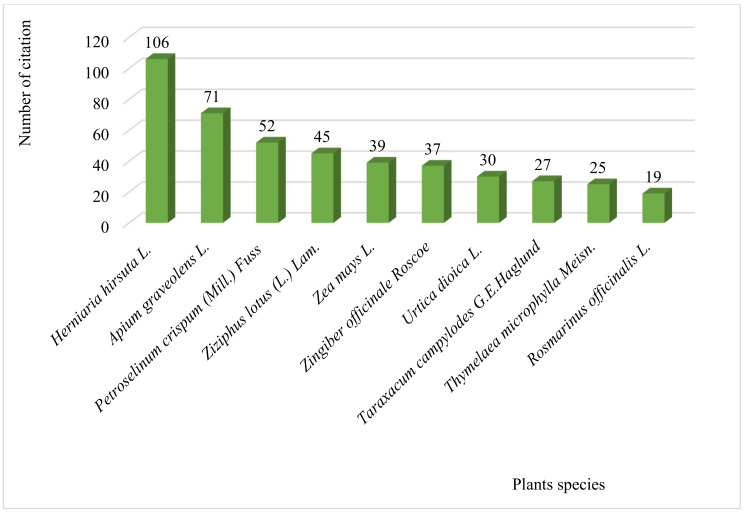
Plant species commonly used traditionally by local people to treatkidney disease.

As shown in Figure 2, the most preferred plant species used to treat kidney diseases in remote areas of North-Eastern Moroccan folk medicine were *H. hirsuta* with (106 use reports; 14.29% of total use reports), followed by *A. graveolens* (71 use reports; 9.57% of total use reports), *P. crispum* (52 use reports; 7.00% of total use reports), and *Z. lotus* (45 use reports; 6.06% of total use reports), *Z. mays* (39 use reports; 5.26% of total use reports), *Z. officinale* (37 use reports; 4.99% of total use reports), *U. dioica* (30 use reports; 4.04% of total use reports), *T. campylodes* (27 use reports; 3.64% of total use reports), *T. microphylla* (25 use reports; 3.37% of total use reports), and *R. officinalis* (19 use reports; 2.57% of total use reports). These ten species accounted for 60.78% of total use reports, and the remaining 101 species represent only 39.22% of total use reports. The frequent use of *H. hirsuta*, *P. crispum*, *Z. lotus*, and *Z. mays* against kidney pain are already confirmed in the results of a study conducted in the Fes-Meknes region of Morocco [19]. These four medicinal plants are widely used in Moroccan folk medicine to manage various diseases [4].

### 2.3. Ethnic Medicinal Characteristics

#### Used Plant Parts and Method of Preparation

**Figure 3:**
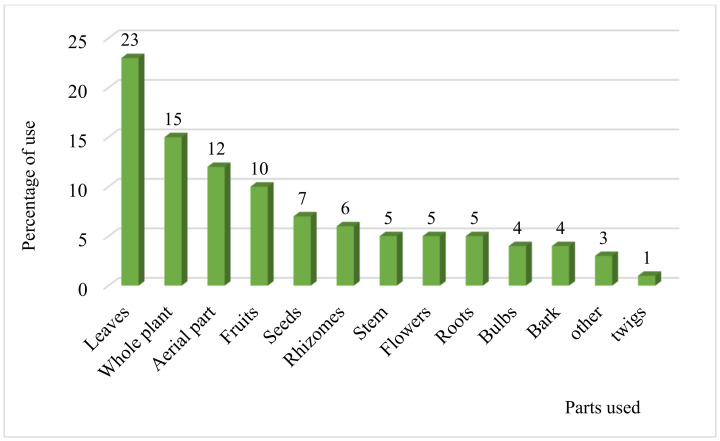
Percentage of the different parts used.

In this survey, several parts of plant species are used as medicine (Figure 3). The most widely used medicinal plant part was the leaves with a frequency of 23%, followed by the whole plant with a percentage of 15%, aerial parts (12%), fruits (10%), seeds (7%), rhizomes (6%) and the other parts (stems, flowers, roots, bulbs, bark, and twig) are represented by a rate lower than 6%. Likewise, several communities in other regions of Morocco and other countries use leaves to prepare herbal medicines [29,30,31]. The frequent use of one part over another in herbal medicine depends on its active ingredient content. The leaves are the most exploited plant parts. This could be explained by the fact that they are both sites of photosynthesis and reservoirs of secondary metabolites that have [32,33]. The rapidity and ease of leaf harvesting also explain their predominance over other plant parts [29]. Besides, harvesting these organs is a relatively sustainable practice compared to other plant parts, such as roots and stem. The harvesting of the roots could contribute to the extermination and disappearance of the plants.

**Figure 4:**
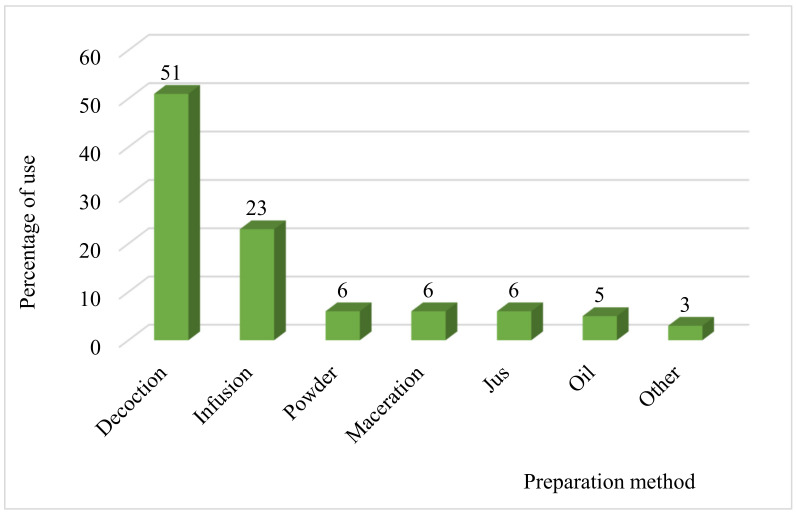
Percentage of different mode of preparation.

As shown in Figure 4, the preparation method most used by the population of North-Eastern Morocco for the treatment of kidney disorders is decoction with a frequency of 51%, followed by infusion (23%), powder, maceration, and juice with a percentage of 6% for each, oil (5%), and other methods of preparation represent only 3%. This high percentage of decoction shows that the local population grows at this mode of preparation and finds it suitable for warming the body and disinfecting the plant [34]. On the other hand, the decoction makes it possible to collect the most active ingredients and attenuates or cancels specific recipes’ toxic effects [35].

### 2.4. Commonly Treated Kidney Diseases and Noteworthy Plants

**Figure 5:**
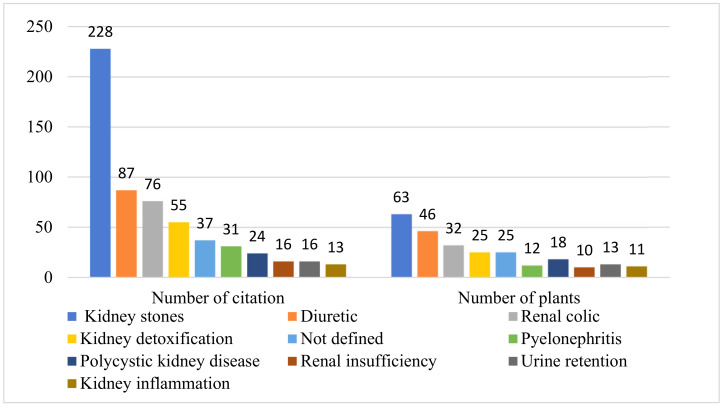
Distribution of plants used traditionally to treat various kidney syndromes.

Traditionally, the local population uses the species inventoried in this survey to treat a wide range of kidney symptoms. Nevertheless, it should be noted that the most mentioned kidneys symptoms (Figure 5) are kidney stones (228 citations, 63 plants), followed by diuretic (87 citations, 46 plants), renal colic (76 citations, 32 plants), kidney detoxification (55 citations, 25 plants) and Pyelonephritis (31 citations, 12 plants). Some species such as *H. hirsuta* (106 use reports), *A. graveolens* (71 use reports), and *P. crispum* (52 use reports) were the most commonly used species for the treatment of kidney symptoms. The aerial parts of *H. hirsuta*, in decoction, are used against kidney stones, the infused leaves are used against Pyelonephritis and renal colic, the whole plant, in decoction, is used to relieve pain in the kidneys, and as well as for detoxifying the kidneys. The aerial part of *A. graveolens*, in decoction, is used against swelling of the kidneys, decocted roots are used to improve the kidneys’ performance, and the infusion of the aerial part against renal colic and kidney stones.

### 2.5. Quantitative Analysis

#### 2.5.1. The Use Value (UV)

The local population’s choice to use certain medicinal species more than others to treat different kidney symptoms is confirmed by the use-value index (UV). The high score of this index reflects the importance of the plant in the study area population. The use-value (UV) results were presented in Table 3, with limited values between 0.16 and 0.0024. According to our results, *H. hirsuta* is the most used by the local population to treat renal disorders with high use value (UV = 0.161), followed by *P. crispum* (UV = 0.114), *Z. lotus* (UV = 0.083), *Z. mays* (UV = 0.052), *Z. officinale* (UV = 0.050), *A. graveolens* (UV = 0.040), *U. dioica* (UV = 0.036), *T. microphylla* (UV = 0.0355), *H. annuus* (UV = 0.034), *T. campylodes* (UV = 0.031), *R. officinalis* (UV = 0.024) and *C. longa* (UV = 0.021). The intensive use of these medicinal species by the population of North-Eastern Morocco is also mentioned with high percentages for the treatment of kidney diseases in the ethnobotanical study conducted in the Northcentral region of Morocco [9], and in other led in the region of Rabat on kidney stones [36].

#### 2.5.2. Botanical Family Use Value (FUV)

As shown in Table 3, the distribution of botanical families of medicinal species in the study area fluctuated between a minimum importance value of 0.0023 and a maximum value of 0.161. Regarding the family use value of the plants recorded in this paper, the results show the high score for Caryophyllaceae (FUV = 0.163), followed by Lamiaceae (FUV = 0.106), Apiaceae (FUV = 0.099), Rhamnaceae (FUV = 0.084), Asteraceae (FUV = 0.083) Poaceae (FUV = 0.074), Asteraceae (FUV = 0.071), Zingiberaceae (FUV = 0.060), Rutaceae (FUV = 0.044), Thymelaeaceae and Urticaceae (FUV = 0.036) for each, Cucurbitaceae (FUV = 0.034) and Ericaceae (FUV = 0.024). The other families have the use value less than 0.024.

#### 2.5.3. Informant Consensus Factor (ICF)

**Figure 6:**
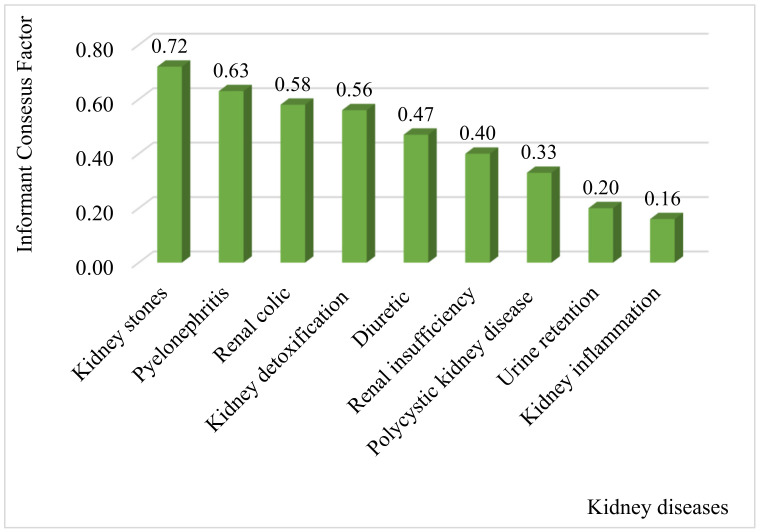
Informant consensus factor (ICF).

The ICF was calculated for each category of renal symptoms, and the index values range from a maximum significance value of 0.72 to a minimum value of 0.16 (Figure 6). Based on these results, we noted that the highest values of this index (ICF) were recorded for kidney stones (ICF = 0.72) with 63 plant species, followed by Pyelonephritis (ICF = 0.63) with 12 plant species, renal colic (ICF = 0.58), kidney poisoning (ICF = 0.56) and diuretic (ICF = 0.47). High values (close to 1) of this index for kidney stones and pyelonephritis indicate that few species were used by a large proportion of informants for each of these two disease categories. For kidney inflammation and urinary retention, the index values were ICF = 0.16 and ICF = 0.22, which means that the number of citations is almost equal to the number of plants used by informants to treat these symptoms. High ICF values for kidney stones may be due to their high incidence of occurrence in the study area [37].

### 2.6. Pharmacological Validation from Literature 

Our ethnobotanical fieldwork indicated that people living in North-Eastern Morocco have important knowledge regarding the use of medicinal plants for the treatment of renal diseases. These ethnobotanical data, which described a wide variety of quantitative indicators, were very interesting for bioprospection purposes. It could be interesting to screen in the literature these plants for their pharmacological activities.

According to the studied literature, among 121 medicinal species inventoried during this survey, 54 plants were studied for their pharmacological properties against kidney disorders, which seems that traditional medicine could be an excellent classical basis for the selection of plant species against kidney problems. The grouped pharmacological data (the plant’s scientific name, the part extracted from the plant; the type of extracts; the experimental model used; the dose used, and the pharmacological effect) of these 54 plants were summarized in Table 4.

**Figure 7:**
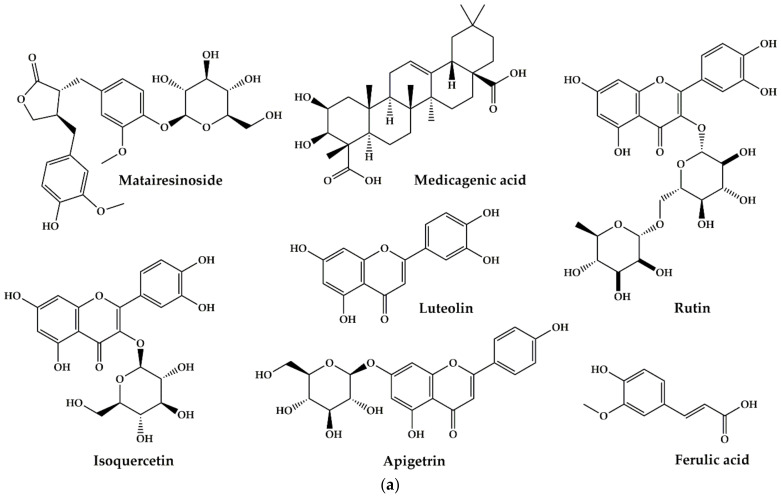

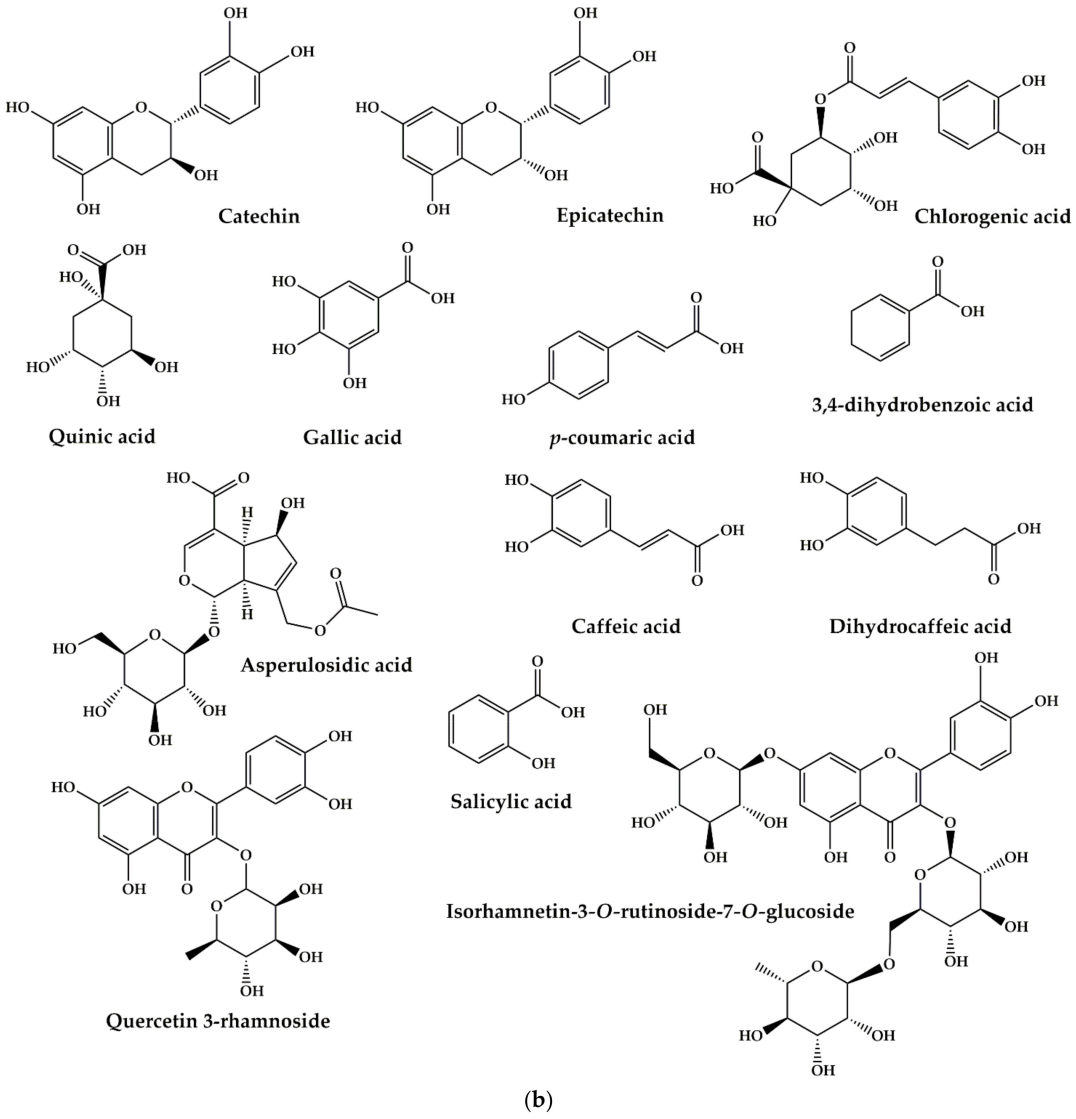
(**a**) Bioactive compounds found in *Herniaria hirsuta L.* extracts; (**b**) Bioactive compounds found in *Herniaria hirsuta* L. extracts.

Among 121 medicinal plants listed in our survey, three plant species, *H. hirsuta*, *A. graveolens*, and P. *crispum* have been the most cited by North-Eastern Morocco people to treat or prevent the traditionally multiple forms of kidneys. In the following paragraphs, we will discuss the potential of these three plants to validate their activity against kidney disorders:

*H. hirsuta* is ranked first as the most cited plant (14.29% of total use reports). According to the traditional knowledge of the North-Eastern Moroccan population, this plant is considered a powerful and common medicinal herb that has shown significant results in treating kidney stones; renal colic; pyelonephritis; kidney pain; diuretic; detoxification of the kidneys; and polycystic kidney disease. From a pharmacological point of view, the aqueous extract of the aerial part of this plant has an inhibitory effect on the crystallization of calcium oxalate in vitro at doses of 0.0625 mg/mL and 0.5% of plant extracts in physiological solution (9 g of NaCl /L) [96,97], and in vivo at a concentration of 50 mg/mL [98], also has an effect on cystine stones in different patients with congenital cystinuria at a dose of 20 g/L [101]. Phytochemical studies have reported and identified some components of *H. hirsuta* include flavonoids, coumarin, tannins and saponins [100,171,172,173]. The active component in the prevention of lithiasis has not yet been identified. However, the literature suggests that the antilithiatic potential of *H. hirsuta* is attributed to saponins with a high probability [171,174]. Recently, a phytochemical study conducted to identify the bioactive constituents of *H. hirsuta* has shown that the aerial part of this plant is rich in phenolic compounds (Figure 7a,b) [171]. According to the literature, these compounds are well known for various pharmacological effects [175,176,177,178,179]. Therefore, the antilithiasic activity of *H. hirsuta* may be due to the presence of these compounds.

**Figure 8:**
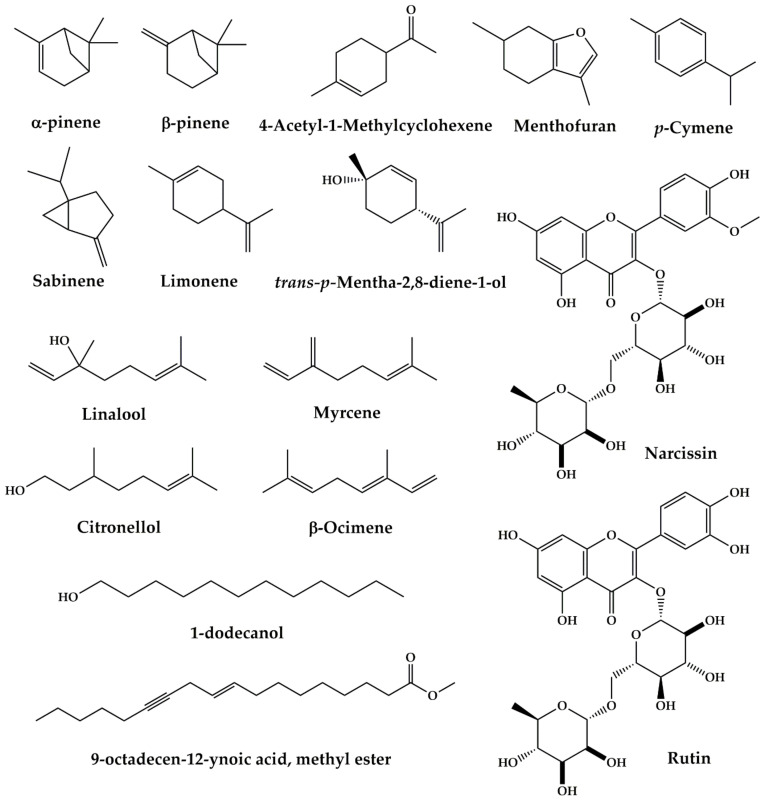
Bioactive compounds from *Apium g.* extracts.

***Apium graveolens* L.** is ranked second, with a percentage of citations of (9.57% of total use reports). It is commonly used to treat several kidney problems: improved kidney performance, kidney swelling, kidney stones, kidney detoxification, kidney pain, diuretic, renal colic, and renal polycystic. The aerial part of *Apium graveolens* L. accentuates urinary excretion of Ca^2+^ in an experimental model of nephron-calcinosis in rabbits at an amount of 8 g/kg added to the animal feed [48]. The ethanolic extract from the stem and leaves of *Apium graveolens* L. demonstrated in vivo a protective effect on kidney damage in the model of rats with ischemia/reperfusion at a dose of 1000 mg/kg body weight [49]. The ethanolic extract and essential oils of fruits of *Apium graveolens* L. have a diuretic effect in vivo in dogs at doses (25 mg/kg; b.w) for the ethanolic extract and (0.004 mL/kg; b.w) for essential oils [50]. The presence of phenolic compounds in the parts of *Apium graveolens* L. is the reason why celery is the plant most used in traditional medicine [180,181]. Previously published photochemical studies have shown that extracts of *Apium graveolens* L. are rich in bioactive compounds such as polyphenols and flavonoids [182,183] (Figure 8). It is well known that these secondary compounds present in *Apium graveolens* L. have considerable pharmacological activities, suggesting that the activities mentioned below may be due to these secondary metabolites.

**Figure 9:**
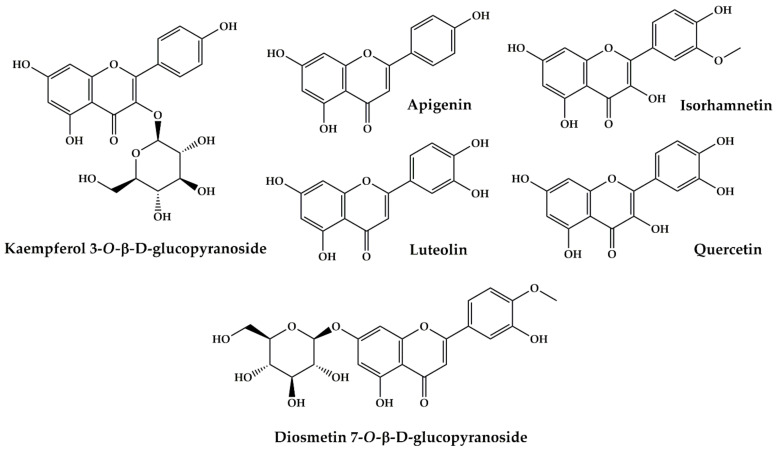
Bioactive compounds from *Petroselinum crispum (Mill.) Fuss* extracts.

*P. crispum* is ranked third as the most cited plant with 7.00% of total use reports. The North-Eastern people of Morocco use this plant against kidney stones, renal colic, and kidney inflammation. The ethanolic extract from the leaves and stem of *P. crispum* has protective effects on acute renal damage induced by ischemia/reperfusion in vivo in rats at doses 100, 150, and 200 mg/kg body weight [133]. At a 200 mg/kg bodyweight concentration, the seeds ethanolic extract showed a protective effect on histopathological changes in the kidneys induced by sodium valproate in male rats [134]. The juice of *P. crispum* has an ameliorative effect against cadmium-induced changes in lipid profile, lipid peroxidation, and catalase activity in the kidneys of *albino* male mice [135]. The aqueous extract from these plant leaves attenuates serum uric acid levels and improves liver and kidney structures in oxolane-induced hyperuricemia rats at doses 3.5, 7.0, 10.5 g/kg of the body weight [136]. Indeed, the pharmacological properties of *P. crispum* are mainly discussed by a wide range of active biomolecules present in this plant. Phytochemical constituents of *P. crispum* were isolated from seeds, roots, leaves or petioles through different separation methods [184]. These phytochemical constituents can be grouped into flavonoids, carbohydrates, coumarins, essential oils and other various compounds. A literature review conducted by Agyare et al. (2017) shows that flavonoids are the most dominant compounds of *P. crispum* such as isorhamnetin, apigenin, quercetin, luteolin, diosmetin 7-O—D-Glucopyranoside, kaempferol 3-*O-β*-d-glucopyranoside (Figure 9) [184]. These phytochemicals may be at the origin of the pharmacological activities of *P. crispum* against the kidney disorders mentioned above.

### 2.7. Constraints of Medicinal Plant’ Uses

Adherence to traditional medical practices by people living in remotes areas of North-Eastern Morocco should be taken with great care. Skepticism, which is, in most cases, based on personal or peer experience regarding the use of medicinal plants, especially the safety and efficacy of the herbal treatment of renal disease. Some medicinal plants’ toxicity, which some users often overlook because of the incorrectly held belief that herbal medications are innocuous, remained critical. The use of medicinal plants faces many problems related to these herbs’ safety that could harm health. This conception was identified as a barrier to some participants that they felt distraught. We know that folk medicine, especially herbal medications, lack the required essential standards of consistency in the pharmacologically active principles of second metabolites containing in these herbs. Besides, the incorrect identification leading to substitution of an innocuous herb, the process of extraction, the adulteration, and the standardization of the use of these herbs contribute to the dangerousness of these herbs.

Several assumptions confirm these. Our team reported that some common plants used as medicine by people in North-eastern Morocco, such as *A. baetica* and *B. dioica*, have evidence of significant concern nephrotoxicity [185]. According to these authors, this toxicity’s causal factors are threefold; the substitution, the misidentification, and the toxic compounds containing in these two species (Aristolochic acid in *A. baetica* and cucurbitacin in *B. dioica*). Another work, published recently by our team reviewed toxic plants in the region, indicated that out of 287 medicinal plants used by local people, 87 plant species had been identified as toxic [186]. In the current work, we found that out of 121 medicinal plant species used traditionally to treat renal diseases, only seven plant species have been identified as toxic by the respondents. The information reported during our interview showed that *E. spinosissimus* and *B. cretica* subsp. Dioica, used separately, could have toxic effects targeting the nervous system leading to excitement and convulsive effects; the consumption of *L. usitatissimum* could have some physiological turbulence such as colic, numbness, and/or respiratory acceleration; *A. succotrina*, when ingested, could have intense organic congestion, eczematous dermatitis, the bulbs of *D. maritima*, in decoction, causes digestive disorders with vomiting, the use of *C. litoralis* subsp. *telephiifolia*. at high dose could have intense diarrhea, and prolonged treatment with the aerial part of *G. glabra* can lead to the digestive system’s neuronal toxicity and disorders. The literature review revealed that among the seven species cited as toxic by the local population, three species were studied for their toxic effects on the laboratory. Hydroethanolic extract of the roots of *C. litoralis* subsp. *telephiifolia*. Showed toxicity in mice with the oral mean lethal dose (LD_50_) value of 14,000 mg/kg body weight [187]. *D. maritima* showed a cytotoxic effect against cancer cells of different lines as in the cell line of non-small cell lung cancer A549 (NSCLC) with IC_50_ = 0.02 µg/mL and in human cervical cancer cell lines Siha and Hela, hosting HPV16 and HPV 18, respectively [188,189]. The aqueous extract of the roots of *B. cretica* subsp. Dioica. showed cytotoxic and apoptogenic activity in Burkitt BL41 lymphoma cell lines at a dose of 125 g/mL [187].

From these observations, we can deduce that although these plants were used traditionally by local people for the treatment of renal diseases and are considered to be safe, for some respondents, they may cause damage due to their unwanted side effects. Therefore, studying medicinal plants’ side effects would have an influential role in identifying and diagnosing the herbs’ safety profile. So, medicinal plants’ consumption without studies of efficacy and safety can result in several side effects that may affect people’s health.

## 3. Materials and Methods

### 3.1. Study Area 

**Figure 10:**
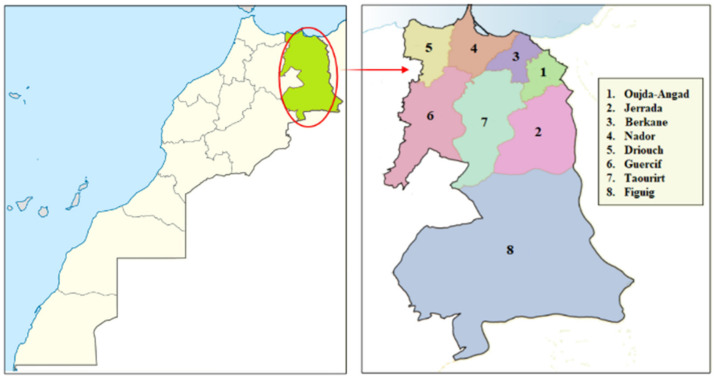
Geographical location of the study area.

The study was conducted in North-Eastern Morocco (Figure 10). This region is limited in the North by the Mediterranean Sea (200 km of coastline), in the East by Moroccan-Algeria fronter, in the south by part of the desert (Figuig province), and in the west by a part of middle Atlas (Taza province). The region includes Benisnassen, Rif, and Horst’s mountainous area, culminating respectively to 1800 m and 1500 m. These geographical features provide the region with a Mediterranean climatic zone that is characterized by hot and dry summers while winters are more cool and wet with average rainfall between 100 mm per year in the South (Saharan bioclimatic zone) and 400 mm per year in the North (Influenced by the Mediterranean Sea). Additionally, the region encompasses several Sites of Biological and Ecological Interest (SBEI) and protected areas such as Benisnassen, Jbel Gorougou. Indeed, these sites had already been identified for their original flora as well as for their biological and ecological qualities [4]. According to the national census conducted in 2014, the region’s total area is 90,130 km^2^, representing 12% of the national territory. Historically, North-Eastern Morocco people have a shared cultural past dating back to the Arab civilization in the seventh century. The cumulative traditional culture, related to ethnobotanical knowledge, has been maintained until now and constitutes the basis for the region’s traditional medical system [6,7].

### 3.2. Ethnobotanical Survey

In order to collect the traditional knowledge about medicinal plants used by people living in the study area, an ethnobotanical survey was conducted from February 2018 to January 2020 in thirteen rural communes of the North-Eastern region of Morocco (Table 2) spread over six provinces (Guercif, Taourirt, Jerada, Berkane, Nador, Oujda-Angad). The ethnobotanical data were randomly selected at thirteen sites visited by conducting semi-structured interviews with 476 respondents from the local population and 12 traditional herbalists. The application of simple random sampling achieved the selection of informants. This sampling technique has the main advantage of ensuring the representativeness of the population. Informants who do not live in the study area are excluded from this study. The questionnaire used consists of two parts: the first one focused on the demographic characteristics of the participants (age, gender, level of education, ethnomedicinal knowledge sources and income of participants…), and the second one focuses on the plant species used in popular medicine for the treatment of kidney disease (vernacular name, parts used, methods of preparation and route of administration).

### 3.3. Identification of Medicinal Plant Species

All local names of plants collected during this study were translated into botanical names, based on the following references [7,190]. For the authentication and the accuracy of plant names listed in this paper of scientific names, we consulted documents specializing in the taxonomy of Moroccan flora (Then, the identification was performed by using standard floras available in Morocco [191,192,193,194,195,196]. For the accuracy and authentication of the scientific nomenclature, the plants recorded were checked against database available online: Catalogue of Life: 2019 Annual Checklist (https://www.catalogueoflife.org/col/) (accessed on 13 April 2020), the Plant List (http://www.theplantlist.org/) (accessed on 13 April 2020) and African Plant Database (http://www.ville-ge.ch/musinfo/bd/cjb/africa/recherche.php) (accessed on 15 April 2020). Only the plant names accepted in these databases were retained. Following the Angiosperm Phylogeny Group IV (2016), the plant families listed in this paper were checked with database APG-IV 2016 [197].

Once the name of each plant species selected was identified correctly, the whole or a part of the picked plants were pressed with a plant press and dried properly. A voucher number was attributed to each specimen and deposited in the Herbarium (HUMPOM), at Mohammed first University, Oujda, Morocco.

### 3.4. Quantitative Data Analysis

#### 3.4.1. Medicinal Use Value (UV)

To give the relative importance of each plant species known locally to be used in popular medicine, we calculated the use-value (UV) for each species. This index was calculated using the following formula [198]:

(1) $$\mathrm{UV}=\frac{\sum U}{N}$$

where UV = use value of species, U = number of quotations per species, N = number of informants.

The value of UV will be higher if there are many reports of use for a plant, which implies that the plant is important, while they will be close to zero if there are few reports related to its use.

#### 3.4.2. Botanical Family Use Value (FUV)

In order to assess the relationship between botanical families and users of species belonging to these families, we used the index called Family Use Value (FUV) which is equal to the average total use value for each species in the family [199].

(2) $$\mathrm{FUF}=\frac{\sum \mathrm{UV}}{N}$$

where FUV = family use value, which equals the average total use value for each species in the family, UV = use value of the species belonging to the family, N = number of species in the family.

#### 3.4.3. Informant Consensus Factor (ICF)

To know about informants’ agreement and consensus, we calculated Index Consensus Factor (ICF) by using the following formula [200]:

(3) $$\mathrm{ICF}=\frac{\left(\mathrm{Nur}-\mathrm{Nt}\right)}{\left(\mathrm{Nur}-1\right)}$$

where Nur is the number of use-reports for a particular ailment category, Nt refers to the number of taxa used for a particular ailment category by all informants.

The ICF values’ margin varies between 0 and 1, where values close to 0 show that the plants are randomly selected or that there is no exchange of information on their use among the informants. Values close to 1 are obtained when there is a well-defined selection criterion within the given community and/or if the information is exchanged between informants.

### 3.5. Bibliographic Review

A review of the available literature on the plants’ biological activities identified against kidney disease was undertaken using the following electronic databases: PubMed, Science Direct, Google Scholar, Scopus, and Web of Science using the following keywords “kidney disease”, “renal disease”, “renal insufficiency”, “nephropathy”, combined with the scientific name of the plant. Chemical structures of plant compounds were performed by Chem Draw 18.1 software.

## 4. Conclusions

This survey showed that people living in North-Eastern Morocco’s remote areas still use medicinal plants to treat ailments, especially renal diseases. The choice of these people was based on their socio-economic and cultural conditions. This preference offered the best chance for them to manage renal sequelae. The people in the study region found that traditional uses of medicinal plants possess suitable healing properties. The results demonstrate the promising role of medicinal plants in managing this particular health problem for these users. However, this preference should be taken with great care. To confirm their therapeutic uses, more investigations are needed to approve the safety and efficacy of their bioactive compounds. Additionally, in predicting the traditionally believed effects of these herbs, researchers need to find out the actuality of their clinical effectiveness and active substances. Once the positive effects of these herbs were proved to be accurate, it is possible to produce drugs useful in the treatment of renal disorders.

## Figures and Tables

**Table 1:** Socio-demographic characteristics of the informants in North-Eastern Morocco.

Distribution of Informants	Categories	Number of Informants	Percentage of Informants (%)
**By sex**	Men	207	42
	Women	281	58
**By age range**	Less than 25 years	42	9
	25–45	132	27
	46–65	260	53
	More than 65 years	54	11
**By education level**	Illiterate	290	59
	Primary education	69	14
	Secondary education	85	17
	University education	44	9
**By income/month**	Unemployed	311	64
	500–2000 DH *	108	22
	2000–6000 DH *	53	11
	>6000 DH *	16	3
**By choice of medicine**	Herbal medicine	264	54
	Both conventional and herbal medicine	161	33
	Modern medicine	63	13

* 1 MAD (Moroccan Dirham) = 0.11 USD (United States Dollar).

**Table 2:** Number of informants for each station.

Provinces	Stations	Number of Informants	
		Population	Herbalist
Guercif	Ras Laksar	22	0
	Saka	43	1
	Jal	25	0
Taourirt	Gteter	37	1
	Debdou	21	1
Jerada	Ain Benimathar	17	1
	Guenfouda	48	2
	Jerada	25	0
Berkane	Naima	18	0
	Tafoughalt	15	2
	Ahfir	14	0
	Chouihia	21	1
Nador	Tiztoutine	29	1
	Bouarg	22	1
	Bni Sidel Jbel	18	0
	Afsou	20	0
Oujda-Angad	Bni Drar	69	1
	Sidi Moussa Lemhaya	12	0
Total	18 stations	476	12

**Table 3:** List of medicinal plants species used by local people for the treatment of kidney diseases.

Scientific Name (Voucher Number)	Local/English Name	Therapeutic Uses	Part Used/Mode of Preparation/Mode of Administration	Common Traditional Dosages	UR	UV	FUV
ALLIACEAE *Allium cepa* L. (HUMPOM628)	البصل/Onion	Renal insufficiency, renal colic, kidney stones, diuretic	bu, st, fr/jui, dec/oral	-	6	0.014	0.017
*Allium sativum* L. (HUMPOM631)	الثوم/Garlic	Renal insufficiency, kidney stones, kidney inflammation, pyelonephritis, polycystic kidney disease	bu/dec	-	2	0.005	
ALOACEAE *Aloe vera* (L.) Burm.f (HUMPOM632)	الالوفيرا/Aloe v.	Renal insufficiency, polycystic kidney disease	wp, ap/jui, dec/oral	Spoon, glass	2	0.005	0.007
*Aloe succotrina* Lam. (HUMPOM629)	الصبار/Fynbos aloe	Renal insufficiency	wp, ap/jui/oral	Spoon, glass	2	0.005	
AMARANTHACEAE *Anabasis aretioides* Moq. and Coss. ex Bunge * (HUMPOM692)	أكنود/Anabasis	Diuretic, polycystic kidney disease	lf/dec/oral	Teapot	1	0.002	0.010
*Beta vulgaris subsp. adanensis* (Pamukç.) Ford-Lloyd and J.T. Williams (HUMPOM630)	باربة/beetroot	Diuretic	rt/mac/oral	Handful	1	0.002	
*Dysphania ambrosioides* (L.) Mosyakin and Clemants (HUMPOM693)	مخينزة/Mexican tea	Diuretic, kidney stones	lf/inf, dec/oral	Handful, Teapot	3	0.007	
ANACARDIACEAE *Pistacia atlantica* Desf. * (HUMPOM694)	لبطم/Atlas mastic tree	Diuretic	cortex/dec/oral	Spoon	1	0.002	0.008
*Pistacia lentiscus* L. * (HUMPOM632)	المسكةالحرة, ذرو/Mastic tree	Diuretic, renal insufficiency, kidney stones	lf/dec, inf/oral	Spoon, handful	3	0.007	
APIACEAE *Daucus carota* L. * (HUMPOM696)	زرودية, خيزو/Wild carrot	Renal pain, diuretic, pyelonephritis	rh/inf/oral	Glass	1	0.002	0.010
*Foeniculum vulgare* Mill. * (HUMPOM697)	النافع/Fennel	Kidney stones, renal colic, renal detoxification	se, lf/inf, tis, dec/oral	Handful, spoon, teapot	8	0.019	
*Petroselinum crispum* (Mill.) Fuss * (HUMPOM695)	المعدنوس, البقدونس/Parsley	Kidney stones, renal colic, renal detoxification Renal pain, diuretic, kidney inflammation, polycystic kidney disease	wp, lf, ap, st, se, rt/inf, mac, dec, oil, jui /oral	Teapot, pinch handful,	**48**	**0.114**	
*Ammi visnaga* (L.) Lam. * (HUMPOM698)	البشنيخة/Toothpick-plant	Kidney stones, renal pain, renal colic, polycystic kidney disease	se, fr, lf/dec, inf, mac/oral	Spoon, glass	7	0.017	
*Ammodaucus leucotrichus* Coss. * (HUMPOM699)	الكمون الصوفي/-	Renal colic, polycystic kidney disease	lf/dec/oral	Handful	1	0.002	
*Apium graveolens* L. * (HUMPOM633)	الكرافس/Celery	Improved kidney performance, kidney swelling, kidney stones, renal detoxification, renal pain, diuretic, renal colic, polycystic kidney disease	rt, tw, ap, lf/inf, dec/oral	Glass, teapot	**17**	0.040	
*Coriandrum sativum* L. * (HUMPOM700)	قصبور/Coriander	Kidney stones, diuretic	wp, ap, lf/inf, dec/oral	Glass, teapot	5	0.012	
*Cuminum cyminum* L. (HUMPOM701)	الكمون/Cumin	Diuretic, kidney stones	lf/inf, dec/oral	Spoon	2	0.005	
*Daucus crinitus* Desf. * (HUMPOM702)	بوزفور/Common carrot	Detoxification of the kidneys	rt/dec/oral	teapot	1	0.002	
*Pimpinella anisum* L. (HUMPOM703)	حبة حلاوة/Aniseed	Diuretic; kidney stones	fr, lf/dec/oral	Spoon	1	0.002	
ASCLEPIADACEAE *Caralluma europaea* (Guss.) N.E.Br. * (HUMPOM634)	الدغموس/Caralluma	Urine retention, kidney stones, polycystic kidney disease	wp, ap/inf/oral	Spoon	2	0.005	0.005
ASPHODELACEAE *Asphodelus microcarpus* Salzm. and Viv. (HUMPOM745)	البروغ/Common asphodel	Diuretic	rt/dec/oral	Handful	1	0.002	0.002
ARALIACEAE *Panax bipinnatifidus* *var. angustifolius* (Burkill) J.Wen (HUMPOM635)	جينسخ/Panax	Diuretic	rh/tis/oral	Spoon	1	0.002	0.002
ASTERACEAE *Echinops spinosissimus* Turra * (HUMPOM704)	تسكرة/Spiny globe thistle	Diuretic, kidney stones, polycystic kidney disease	ap, rt/inf, dec/oral	Spoon, teapot	5	0.019	0.083
*Helianthus annuus* L. (HUMPOM636)	نوار الشمس/Sunflower	Renal pain, kidney inflammation	se, fl/dec, inf, mac/oral	Spoon	**14**	0.033	
*Lactuca sativa* L. (HUMPOM637)	خس/Lettuce	Kidney inflammation, polycystic kidney disease	lf/mac/oral	Spoon	1	0.002	
*Artemisia arborescens* (Vaill.) L. (HUMPOM638)	الشيبة/Tree wormwood	Kidney stones, renal colic, renal detoxification, diuretic, renal colic, pyelonephritis, polycystic kidney disease	lf/dec, inf, mac/oral	Teapot, glass	6	0.014	
*Artemisia campestris* L. (HUMPOM705)	ألاال/Wormwood sagewort	Kidney stones	lf/dec/oral	Spoon	1	0.002	
*Brocchia cinerea* (Delile) Vis. (HUMPOM706)	قرطوفة/-	Kidney stones	lf/mac/oral	Spoon	1	0.002	
*Cichorium intybus* L. * (HUMPOM707)	بوعكاد/Common chicory	Diuretic	rt/dec/oral	Spoon	1	0.002	
*Cynara cardunculus* L. (HUMPOM709)	الخرشف/Cardoon	Pyelonephritis	rt/pow/oral	Handful	1	0.002	
*Dittrichia viscosa* (L.) Greuter * (HUMPOM708)	مكرمان/False yellowhead	Kidney stones, pyelonephritis	wp/dec/oral	Handful	1	0.002	
*Glebionis coronaria* (L.) Cass. ex Spach * (HUMPOM710)	رجل لفلوس/Garland chrysanthemum	Kidney stones	wp/inf/oral	Handful	1	0.002	
*Rhaponticum acaule* (L.) DC. (HUMPOM712)	التابغة/Maral root	Renal detoxification, renal pain	rt/dec/oral	Glass	1	0.002	
*Scorzonera undulata* Vahl (HUMPOM711)	التالمة/Viper’s grass	Renal detoxification	rt/dec/oral	Spoon	1	0.002	
*Taraxacum campylodes* G.E.Haglund * (HUMPOM639)	الهندباء/Common dandelion	Renal detoxification, kidney stones, kidney inflammation, pyelonephritis, diuretic	wp, lf, se/inf, dec/oral	Spoon, teapot, glass	**13**	0.030	
*Tanacetum cinerariifolium* (Trevir.) Sch.Bip (HUMPOM713)	عود العطاس/Pyrethrum	Kidney stones	st/inf/oral	Spoon	1	0.002	
BERBERIDACEAE *Berberis vulgaris subsp. australis* (Boiss.) Heywood * (HUMPOM714)	إرغيس/Common barberry	Kidney stones	st/pow/oral	Spoon	1	0.002	0.002
BORAGINACEAE *Borago officinalis* L * (HUMPOM715)	الحريشة/Burrage	Diuretic	lf/dec/oral	Spoon	1	0.002	0.002
BRASSICACEAE *Brassica napus* L. (HUMPOM640)	الفت/Annual rape	Diuretic	ap/dec/oral	Spoon	1	0.002	0.006
*Brassica oleracea* L. (HUMPOM641)	لكروم/Wild cabbage	Renal pain	lf/dec/oral	Handful	1	0.002	
*Lepidium sativum* L. (HUMPOM642)	حب الرشاد/Common cress	Urine retention	se/dec/oral	Spoon	1	0.002	
BURSERACEAE *Boswellia ameero* Balf.f. (HUMPOM716)	لبان ذكر/ Socotra Frankincense Tree	Pyelonephritis	se/dec/oral	Spoon	1	0.0024	0.0024
CACTACEAE *Opuntia ficus-indica* (L.) Mill. (HUMPOM717)	الهندية/ Prickly Pear	Diuretic, kidney stones	fl, lf, fr/dec, mac/oral	Spoon	3	0.007	0.007
CAESALPINIACEAE *Ceratonia siliqua* L. (HUMPOM118)	الخروب, تسلغو/ Carob	Renal insufficiency, renal colic, kidney stones	fr/dec, pow/oral	Spoon, handful	3	0.007	0.007
CARYOPHYLLACEAE *Corrigiola litoralis* *subsp. telephiifolia* (Pourr.) Briq. * (HUMPOM719)	سرغينة/Strapwort	Diuretic	wp/dec/oral	Handful	1	0.002	**0.162**
*Herniaria hirsuta* L. * (HUMPOM730)	هراسة لحجر/Hairy rupturewort	Kidney stones, renal colic, pyelonephritis, renal pain, diuretic, renal detoxification, polycystic kidney disease	wp, ap, st, lf/inf, dec/oral	Handful, Spoon, teapot, glass	**68**	**0.161**	
CONVOLVULACEAE *Convolvulus althaeoides* L. * (HUMPOM720)	اللواية/Mallow bindweed	Kidney stones, polycystic kidney disease	se/pow/oral	Handful	2	0.005	0.005
CUCURBITACEAE *Citrullus lanatus* (Thunb.) Matsum. and Nakai (HUMPOM643)	الدلاح, الدليع/Watermelon	Urine retention, renal colic, renal detoxification, renal insufficiency, polycystic kidney disease	ba, fr/inf, jui, dec/oral	Spoon, glass	6	0.014	0.034
*Bryonia cretica* subsp. dioica (Jacq.) Tutin (HUMPOM644)	عنب الديب/Bryony	Kidney inflammation	fr/dec/oral	Glass	1	0.002	
*Cucumis melo* L. (HUMPOM645)	بتيخ/Honeydew	Renal pain	fr/eat/oral	-	1	0.002	
*Cucumis sativus* L. (HUMPOM646)	خيار/Cucumber	Renal pain	fr/jui/oral	Glass	1	0.002	
*Cucurbita pepo* L. (HUMPOM647)	الكارعة/Pumpkin	Kidney stones, urine retention, renal pain, diuretic	se, lf/inf, dec/oral	Spoon	6	0.014	
CUPRESSACEAE *Juniperus oxycedrus* L. * (HUMPOM721)	تيغا/Prickly juniper	Kidney stones, renal colic	lf/dec/oral	Spoon	5	0.012	0.015
*Tetraclinis articulata* (Vahl) Mast. * (HUMPOM722)	العرعار/Arar tree	Renal colic, kidney stones, diuretic	lf/dec/oral	Spoon, handful	3	0.007	
EQUISETACEAE *Equisetum arvense* L. (HUMPOM746)	عشبةذيلالحصان/Field horsetail	Renal colic, kidney stones	ap/dec, inf/oral	Spoon	3	0.007	0.005
ERICACEAE *Vaccinium macrocarpon* Aiton (HUMPOM747)	التوت البري/Cranberry	Kidney stones, renal insufficiency, diuretic	fr/mac, dec/oral	Glass	5	0.012	0.024
*Arbutus unedo* L. * (HUMPOM748)	ساسنو/Strawberry tree	Renal pain, diuretic, renal colic, polycystic kidney disease	rt, lf/dec/oral	Spoon, handful	5	0.012	
EUPHORBIACEAE *Euphorbia retusa* Forssk (HUMPOM723)	تنورا/Spurge	Kidney stones	lf/inf/oral	Handful	1	0.002	0.002
FABACEAE *Anagyris foetida* L. * (HUMPOM648)	فول الكلب/Stinking bean trefoil	Kidney stones	se/inf/oral	Handful	1	0.002	0.022
*Arachis hypogaea* L. (HUMPOM649)	القاوقاو/Peanut	Urine retention	ba, se/dec, mec/oral	Handful	2	0.005	
*Glycyrrhiza glabra* L. (HUMPOM650)	عرق السوس/Lecorice	Renal colic, diuretic, renal pain	rt, st/inf, dec, mac/oral	Teapot	7	0.017	
*Trigonella foenum-graecum* L. (HUMPOM725)	الحلبة/Spice fenugreek	Improved kidney performance, renal pain, diuretic	se/inf, dec, mac/oral	Spoon	4	0.010	
*Vicia faba* L. (HUMPOM724)	الفول/Broad bean	Renal pain	se/dec/oral	Handful	1	0.002	
FAGACEAE *Quercus suber* L. * (HUMPOM651)	الدباغ/Cork oak	Kidney stones	lf, ba/dec/oral	Spoon	1	0.002	0.002
GENTIANACEAE *Centaurium erythraea* Rafn * (HUMPOM726)	كوزة الحية/Common centaury	Renal pain	ap/dec/oral	Handful	1	0.002	0.002
GLOBULARIACEAE *Globularia alypum* L. * (HUMPOM728)	تسلغا/Alypo globe daisy	Kidney stones, pyelonephritis	lf/dec/oral	Spoon, handful	4	0.010	0.010
HYACINTHACEAE *Drimia maritima* (L.) Stearn * (HUMPOM729)	بصلة لخلا/Maritime squill	Diuretic, kidney stones, urine retention	bu/inf, dec, mac/oral	Glass	4	0.010	0.010
IRIDACEAE *Crocus sativus* L. (HUMPOM652)	الزعفران/Saffron	Kidney stones, diuretic, renal colic, kidney inflammation, polycystic kidney disease	ap, sta/pow, dec, inf/oral	Pinch	6	0.014	0.014
JUNCACEAE *Juncus acutus* L. * (HUMPOM731)	سمار، أزلاف/Spiny rush	Diuretic	wp/inf/oral	Spoon	1	0.002	0.002
LAMIACEAE *Ajuga iva* (L.) Schreb. * (HUMPOM653)	شندكورة/Southern bugle	Renal detoxification, kidney stones	ap/dec, inf/oral	Handful	2	0.005	**0.107**
*Clinopodium nepetasubsp. glandulosum* (Req.) Govaerts * (HUMPOM654)	مانتة/Lesser calamint	Renal colic, diuretic	lf, st, ap/oin, mac, dec, inf/mas, oral	Handful, teapot	7	0.017	
*Lavandula dentata* L. * (HUMPOM655)	الخزامة/French lavender	Kidney swelling, urine retention, renal detoxification	lf, wp, fl/dec, inf/oral	Spoon	7	0.017	
*Mentha pulegium* L. * (HUMPOM656)	فليو/Pennyroyal	Renal colic, kidney stones	wp, ap/dec, inf/oral	Teapot	2	0.005	
*Mentha spicata* L. (HUMPOM657)	نعناع/Mint	Renal pain	lf/dec/oral	Teapot	1	0.002	
*Mentha suaveolens* Ehrh. * (HUMPOM658)	تمرصاد/Bigleaf mint	Kidney inflammation	lf/pow/oral	Spoon	1	0.002	
*Ocimum basilicum* L. (HUMPOM659)	لحبق/Sweet basil	Renal pain	lf/inf/oral	Spoon	1	0.002	
*Origanum compactum* Benth. * (HUMPOM660)	الزعتر/Oregano	Renal colic, kidney swelling, urine retention, pyelonephritis, renal pain, polycystic kidney disease	lf, ap/inf, dec, tis/oral	Spoon, teapot	5	0.012	
*Origanummajorana* L. (HUMPOM661)	البرددوش/Sweet marjoram	Renal colic, renal pain, urine retention, pyelonephritis	lf/inf, dec/oral	Handful	2	0.005	
*Rosmarinus officinalis* L. * (HUMPOM662)	أزير/Rosemary	Kidney stones, kidney inflammation, renal detoxification, urine retention, renal colic, diuretic, renal pain, polycystic kidney disease	lf, ap/inf, dec/oral	Teapot	**10**	0.024	
*Salvia officinalis* L. (HUMPOM663)	السالمية/Sage	Kidney stones, diuretic, renal colic	ap, lf/inf, dec/oral	Handful, spoon	7	0.017	
*Thymus saturejoides* Coss. * (HUMPOM664)	أزوكني/Thyme	Kidney inflammation	lf/inf, dec/oral	Handful	1	0.002	
LAURACEAE *Laurus nobilis* L. * (HUMPOM732)	ورقسيدناموسى/Bay tree	Renal colic, kidney stones	lf/dec, inf/oral	Handful	2	0.005	0.010
*Cinnamomum cassia* (L.) J.Presl (HUMPOM733)	القرفة/Chinese cassia	Kidney stones	ba/pow/oral	Spoon	3	0.007	
LINACEAE *Linum usitatissimum* L. (HUMPOM734)	زريعة الكتان/Flaxseed	Renal diseases, diuretic	se/dec/oral	Spoon	1	0.002	0.002
LYTHRACEAE *Lawsonia inermis* L. (HUMPOM665)	الحنى/Mignonette tree	Kidney stone	lf/dec/oral	Spoon	1	0.002	0.002
MORACEAE *Morus alba* L. (HUMPOM735)	التوت/White mulberry	Renal colic, diuretic, renal detoxification	lf, fr/mac, dec, inf/oral	Glass	4	0.010	0.010
MYRTACEAE *Myrtus communis* L. (HUMPOM666)	الريحان/Common myrtle	Renal detoxification, pyelonephritis	lf/dec/oral	Spoon	1	0.002	0.020
*Eucalyptus globulus* Labill. (HUMPOM667)	الكاليتوس/Tasmanian blue gum	Renal colic	lf/dec/oral	Handful, teapot	4	0.010	
*Syzygium aromaticum* (L.) Merr. and L.M.Perry (HUMPOM668)	القرنفل/Clove	Renal insufficiency, renal colic, renal pain	ap, lf/dec, inf/oral	Spoon	4	0.010	
*OLEACEAE* *Fraxinus excelsior* L. (HUMPOM737)	لسان طير/Common ash	Kidney stones, pyelonephritis, polycystic kidney disease	lf/inf/oral	Handful, spoon	4	0.010	0.007
*Olea europaea* L. (HUMPOM736)	الزيتون/Olive	Renal detoxification, kidney stones, diuretic	lf, fr/dec, oil/oral	Spoon (oil), handful	3	0.007	
PAPAVERACEAE *Papaver rhoeas* L. * (HUMPOM669)	بنعمان/Common poppy	Kidney stones, kidney inflammation, pyelonephritis	se/pow/oral	Handful	1	0.002	0.002
PIPERACEAE *Piper cubeba* L. f. (HUMPOM670)	كبابة/Cubeb pepper	Pyelonephritis, polycystic kidney disease	fr/inf/oral	Handful	1	0.002	0.002
PLANTAGINACEAE *Globularia repens* Lam. (HUMPOM671)	عين لرنب/Creeping globe daisy	Renal insufficiency, kidney stones, urine retention	lf/pow/oral	Spoon	3	0.007	0.007
POACEAE *Pennisetum glaucum* (L.) R.Br. (HUMPOM738)	إيلان/Yellow bristlegrass	Renal pain, polycystic kidney disease, pyelonephritis	se/pow/oral	Spoon	1	0.002	0.074
*Avena sativa* L. (HUMPOM741)	الخرطال/Common oat	Diuretic, renal pain	se/dec/oral	Handful	1	0.002	
*Cynodon dactylon* (L.) Pers. * (HUMPOM740)	عروق النجم/Bermuda grass	Kidney stones, renal pain, diuretic	rt, lf/dec, mac, inf/oral	Handful	3	0.007	
*Hordeum vulgare* L. (HUMPOM739)	شعير/Barley	Diuretic, kidney stones	fr, se/dec, mac/oral	Handful	2	0.005	
*Zea mays* L. (HUMPOM742)	الذرة/Maize	Kidney stones, kidney swelling, renal insufficiency, renal pain, diuretic, kidney inflammation, polycystic kidney disease	fr, fl/dec, inf/oral	Handful	**22**	0.052	
POLYGONACEAE *Rumex vesicarius* L. (HUMPOM672)	حميضة/Ruby dock	Renal detoxification, kidney stones	ap, lf/dec, inf/oral	Handful	2	0.005	0.007
PUNICACEAE *Punica granatum* L. (HUMPOM743)	الرمان/Pomegranate	Renal detoxification, renal colic, kidney stones, renal pain, pyelonephritis	ba, fr/dec, pow/oral	Glass	5	0.012	0.012
RANUNCULACEAE *Nigella sativa* L. (HUMPOM744)	السانوج, حبةالكحلة/Black caraway	Detoxification of the kidneys, diuretic	se/dec, inf, oil/oral	Pinch	3	0.007	0.007
RHAMNACEAE *Ziziphus jujuba* Mill. (HUMPOM673)	زفيزف/Lotus jujube	Renal detoxification, pyelonephritis	fr/inf/oral	Handful	1	0.002	0.084
*Ziziphus lotus* (L.) Lam. (HUMPOM674)	السدرة، النبق/Lotus tree	Urine retention, diuretic, renal colic, kidney stones, pyelonephritis, polycystic kidney disease	rt, fr, lf/dec, inf, pow/oral	Handful, spoon	**35**	0.083	
ROSACEAE *Malus sylvestris* (L.) Mill. (HUMPOM675)	التفاح/Common apple	Kidney swelling, renal colic, kidney stones	fr/inf, eat/oral	-	5	0.012	0.020
*Prunus cerasus* L. (HUMPOM677)	حب لملوك/Sour cherry	Renal colic, renal pain, diuretic, kidney stones	tw, fr/dec/oral	Handful	3	0.007	
*Rosa canina* L. * (HUMPOM676)	الورد البلدي/Common briar	Diuretic, pyelonephritis	lf/mac/oral	Handful	1	0.002	
RUBIACEAE *Rubia peregrina* L. (HUMPOM678)	الفوة/Common wild madder	Renal pain, kidney stones, diuretic	lf, ap/dec, inf/oral	Handful	4	0.010	0.010
RUTACEAE *Citrus × aurantium* L. (HUMPOM690)	الرنج/Lime	Kidney stones, renal pain	fr/jui/oral	Glass	2	0.005	0.044
*Citrus limon* (L.) Osbeck (HUMPOM691)	اليم/Lemon	Kidney stones, renal insufficiency, renal detoxification,	bu, fr/jui, inf/oral	Glass	6	0.014	
*Citrus saliaefolius* L (HUMPOM688)	وظمي/Sage-leaved rock-rose	Renal detoxification	rt/dec/oral	Handful	1	0.002	
*Citrus sinensis (L.) Osbeck* (HUMPOM689)	الليمون/Sweet orange	Renal colic, renal insufficiency, kidney stones	fr/jui, inf/oral	Glass	6	0.014	
*Ruta montana (L.) L.* * (HUMPOM687)	أورمي/Rue	Kidney stones, polycystic kidney disease	lf/inf/oral	Spoon	1	0.002	
SOLANACEAE *Capsicum annuum* L. (HUMPOM679)	الحار/Capsicum pepper	Diuretic	fr/dec, pow/oral	Pinch	2	0.005	0.005
THYMELAEACEAE *Thymelaea microphylla* Meisn. * (HUMPOM680)	المتنان/Sparrow-worts	Diuretic, renal colic, kidney stones, pyelonephritis	lf, ap/dec, mac/oral	Handful, teapot	**14**	0.005	0.036
TILIACEAE *Tilia sylvestris* Desf (HUMPOM681)	زيزفون/Small-leaved linden	Kidney stones, renal detoxification	ap, lf/dec/oral	Glass	1	0.002	0.010
URTICACEAE *Urtica dioica* L. * (HUMPOM682)	الحريكة الملساء/Common nettle	Urine retention, kidney stones, diuretic, renal insufficiency, renal pain, kidney swelling, pyelonephritis, renal colic, kidney inflammation	st, ap, wp, lf/dec, pow, inf/oral	Handful	**15**	0.036	0.036
VERBENACEAE *Aloysia citriodora* Palau (HUMPOM683)	اللويزة/Lemon verbena	Diuretic, pyelonephritis	lf/dec, inf/oral	Handful	3	0.007	0.007
VITACEAE *Vitis vinifera* L. (HUMPOM684)	الدالية/Wine grape	Renal detoxification, diuretic, pyelonephritis	lf/dec	Glass	3	0.007	0.007
ZINGIBERACEAE *Curcuma longa* L. (HUMPOM685)	الخرقوم/Turmeric	Renal detoxification, renal colic, kidney stones	rh/pow, dec, inf/oral	Spoon	8	0.019	0.060
*Zingiber officinale* Roscoe (HUMPOM686)	الزنجبيل,سكينجبير/Ginger	Kidney swelling, kidney stones, renal detoxification, detoxification of the kidneys, kidney inflammation, renal pain, diuretic, polycystic kidney disease	rh, rt/pow, dec, inf/oral	Spoon, pinch	**19**	0.045	

Abbreviation: parts used: **bu:** bulb; **st**: stem; **fr**: fruit; **wp**: whole plant; **ap**: aerial part; **lf**: leaf; **rt**: root; **rh**: rhizome; **se**: seeds; **tw**: twigs; **fl**: flowers; **ba**: bark; **sta**: stamen. Mode of preparation: juice: **jui**; decoction: **dec**; infusion: **inf**; maceration: **mac**; powder: **pow**; tisane: **tis**; ointment: **oin**; massage: **mas**; **UV**: Use Value. **FUV**: Family Use Value. **UR**: Use reports. *: Endemic.

**Table 4:** Pharmacological data of the medicinal species cited by local people to treat kidney diseases.

Scientific Name	Used Parts	Used Extracts	Experimental Model	Pharmacological Uses	Therapeutic Doses	References
*Ajuga iva* (L.) Schreb.	Whole plant	Aqueous extract	Rats	Beneficial for correcting the hyperglycemia and preventing diabetic complications in liver, pancreas and kidneys	50 mg/kg of body weight daily for 3 weeks	[38]
*Allium sativum* L.	Bulbs	Aqueous extract	Rats	Modulatory effects on renal oxidative stress and nitric oxide production in streptozotocin-induced diabetic nephropathy in rats	200–400 mg/kg of body weight for 30 consecutive days	[39]
	Bulbs	Aqueous extract	Rats	Modulates the expression of angiotensin II AT2 receptor in adrenal and renal tissues of streptozotocin-induced diabetic rats	500 mg/kg of body weight 8 weeks after diabetes induction	[40]
	Bulbs	Aqueous extract	Rats	Protects hepatic and renal toxicity of alloxan in rats	100–200 mg/kg of body weight/day; given by oral gavage for 21 days	[41]
	Bulbs	Ethanol extract	Rats	Ameliorative effects on renal parenchyma of gentamicin-induced nephropathic rats	200 mg/kg of body weight for 10 days	[42]
*Aloe vera* (L.) *Burm.f.*	Leaves	Leaf pulp extract	Rats	Protective effect on mild damage caused by type II diabetic on kidney tissue	500 mg/kg of body weight	[43]
	Leaves	Ethanol extract	Rats	Protective role on liver and kidney of streptozotocin-induced diabetic rats	300 mg/kg of body weight for 30 days	[44]
	Leaves	Ethanol extract	Rats	Antinephropathy effect on PKC-β level of rat kidney in diabetes mellitus	30–120 mg/kg of body weight	[45]
*Ammi visnaga* (L.) Lam.	Fruits	Aqueous extract	LLC-PK1and Madin-Darby-canine kidney (MDCK) cells	Prevent cell damage caused by oxalate in renal epithelial cells	(100 µg/mL)	[46]
	Fruits	Aqueous extract	Rats	Prevention of renal crystal deposition	125–500 mg/kg of body weight for 14 days	[47]
*Apium graveolens* L.	Aerial parts	Fresh celery	Rabbits	Accentuates urinary Ca^+2^ excretions in experimental model of nephrocalcinosis	8 g/kg added to the animal food	[48]
	Stem, leaves	Ethanolic extract	Rats	Protective effect on kidney damage in ischemia/reperfusion injury rats model	250–1000 mg/kg of body weight for 14 days	[49]
	Fruits	Essential oil	Dogs	Diuretic effect	0.004–0.008 mL/kg of body weight	[50]
*Arachis hypogaea* L.	Peanuts pods	Methanol and aqueous extracts	Mice	Nephroprotective effect on CCl_4_ induced kidney damage in mice	50–100 mg/kg of body weight	[51]
*Arbutus unedo* L.	Leaves	Aqueous extracts	Rats	Prevent cardiovascular and renal hemodynamic effects in L-NAME-induced hypertensive rats	250 mg/kg of body weight/day	[52]
*Artemisia arborescens* (Vaill.) L.	Leaves	Hydroalcoholic extract	Rats	Nephroprotective effects against oestroprogestative-induced kidney damages in rats	200 mg/kg body weight during 6 weeks	[53]
*Artemisia campestris* L.	Aerial parts	Essential oil	Rats	Protective effect on Deltamethrin induced oxidative stress in kidney and brain of rats	200 mg/kg of body weight for two weeks	[54]
*Avena sativa* L.	Seeds	Powder	Human	Beneficial effect on serum albumin and serum potassium in patients with CKD	50 g of oat flour per day for 8 weeks	[55]
	Seeds	Seeds prepared with food pellets and distilled water to get a cohesive paste	Mice	Protective effects of against oxidative stress-induced kidney damage resulting from an estrogen deficiency in ovariectomized swiss mice model	200 mg/kg of body weight	[56]
*Berberis vulgaris subsp. australis* (Boiss.) Heywood	Bark	Ethanolic extract	Rats	Ameliorative effects on lipid profile, kidney and liver function in experimental dyslipidemia	300–500 mg/kg of body weight for eight weeks	[57]
*Brassica oleracea* L.	Broccoli sprouts	Juice	Rats	Protective effects toward renal damage in high-salt-fed SHRSP: role of AMPK/PPARa/UCP2 axis	340 mL/120 mg in diet	[58]
*Ceratonia siliqua* L.	Pulp and seeds	Aqueous extract	Rats	Protective effect against a dextran sulfate sodium-induced alteration in liver and kidney in rat	50 and 100 mg/kg of body weight for 21 days	[59]
	Leaves	Ethyl acetate fraction	Rats	Ameliorative effects against CCl4 induced hepatic oxidative damage and renal failure in rats	250 mg/kg of body weight for 8 days	[60]
*Cichorium intybus* L.	Seeds	Aqueous extract	Rats	Improving effect on renal parameters in experimentally induced early and late diabetes type 2 in rats	125 mg/kg of body weight for 21 days	[61]
	Aerial parts	Ethanol extract	Rats	Against cisplatin induced renal toxicity	500 mg/kg of body weight for 10 consecutive days	[62]
	Flowers	Aqueous extract	Rats	Preventive effects on ethylene glycol-induced renal calculi in rats	50–200 mg/kg of body weight for 30 days	[63]
	Roots	Aqueous extract	Rats	Improving effects on serum oxidative stress, liver and kidney volume, and cyclin B1 and Bcl-2 levels in the brains of rats with ethanol induced damage	200 mg/kg of body weight for 18 days	[64]
	Roots	Unspecified	Rats	Ameliorates hydroxyapatite nanoparticles induced kidney damage in rats	20 and 300 mg/kg of body weight for 4 weeks	[65]
*Cinnamomum cassia* (L.) J.Presl	Bark	Methanol extract	Rats	Ameliorative effect against Ni-NPs-induced liver and kidney damage in male Sprague Dawley rats	175–225 mg/kg of body weight	[66]
*Citrus sinensis* (L.) Osbeck	Leaves	Essential oil	Rats	Ameliorative effect on some liver and kidney function indices of diabetic rats	110 mg/Kg of body weight for 15 days	[67]
	Stems	Aqueous and methanolic extracts	Human Embryonic Kidney Carcinoma (HEK) cell line	Anti-proliferative or cytopathic potential effects against human embryonic kidney carcinoma cell line	IC_50_ at 32-fold dilution of the extract	[68]
*Coriandrum sativum* L.	Seeds	Aqueous and ethanol extracts	Mices	Protective role against lead nitrate induced oxidative stress and tissue damage in the liver and kidney in mal mice	WE (300 and 600 mg/kg of body weight), EtOH (250 and 500 mg/kg of body weight)	[69]
*Crocus sativus* L.	Unspecified	Aqueous extract	Cats	Increase the glomerular filtration rate and shortened the emptying half-time of radiopharmaceutical	90 mg/kg body weight	[70]
	Saffron threads	Aqueous extract	Rats	Protect the kidney and liver of diabetic rats against damage caused by hyperglycemia-induced inflammation, due to its anti-inflammatory potential	200 mg/kg of body weight	[71]
	Petals	Hydroalcoholic extract	Rats	Beneficial for the kidneys	200–600 mg/kg of body weight/day	[72]
	Petals	Hydroalcoholic extract	Rats	Protects the kidney	167.5 and 335 mg/kg of body weight/day	[73]
*Cucumis melo* L	Seeds	Ethanolic extract	Mice	Renoprotective effects in gentamicin-induced renal damage	250–500 mg/kg of body weight for 8 days	[74]
	Leaves	Ethanol extract	Rats	Potential and effective role in inhibiting inflammation and oxidative stress in the kidney of diabetic rats	30–120 mg/kg of body weight for 30 consecutive days	[75]
*Cucumis sativus* L.	Pulp	Ethanol extract	Rats	Ameliorative effect on alloxan-induced kidney toxicity in male adult Wistar rats	100–500 mg/kg of body weight for 28 days	[76]
*Cucurbita pepo* L.	Seeds	Methanol extract	Rats	Antiurolithic against sodium oxalate-induced renal calculi	In vivo (250–1000 mg/kg of body weight), in vitro (20–80 mg/mL)	[77]
*Curcuma longa* L.	Rhizomes	Ethanol extract	Rats	Effect on antioxidant enzymes in kidney of alloxan induced type-1 diabetic male rats	250 mg/kg of body weight	[78]
	Rhizomes	Hydro-alcoholic extract	Rats	Protective effect on adriamycin-induced oxidative stress in kidney rat	1000 mg/kg of body weight	[79]
	Rhizomes	Ethanol extract	Chickens	Effect on biochemical and pathological parameters of liver and kidney in chicken aflatoxicosis	5 mg mixed with 1 kg of diet	[80]
	Rhizomes	Polyphenol extract	Rats	Effect on doxorubicin-induced kidney injury in rats	5 mg mixed with 1 g of died	[81]
*Cynodon dactylon* (L.) Pers.	Whole plant	Aqueous extract	Rats	Against kidney stones	12.5, 50 and 200 mg/kg of body weight	[82]
*Daucus carota* L.	Seeds	Methanol extract	Rats	Antihyperlipidemic properties et protective effect on liver and kidney function in diabetic rats	100–300 mg/kg of body weight for 6 days using gavage)	[83]
	Roots	Petroleum ether and methanol extract	Rats	Protective and curative potential on renal ischemia reperfusion injury in rats	250–500 mg/kg of body weight for 14 days	[84]
	Carrot tuber	Aqueous extract	Rats	Hepatoprotective, hepatocurative and nephro-curative properties and could be explored in nutrition and health	300 mg/kg of body weight for 6 weeks	[85]
*Eucalyptus globulus* Labill.	Leaves	Methanol extract	Mice	Hepato–renal protective potential againstCyclophosphamide induced toxicity in mice	50–100 mg/kg of body weight for 15 days	[86]
	Leaves	Aqueous-ethanol extracts	Rats	Protective effect against acetaminophen-induced kidney damages in male rat	130 mg /kg of body weight/day; for 42 days	[87]
*Foeniculum vulgare* Mill.	Fruits	Aqueous extract	Rats	Inhibition of calcium oxalate renal crystals formation in rats	4 mL/100 g body weight for 4 weeks	[88]
	Seeds	Aqueous extract	Rats	Protect liver, kidney and gonadal functionsagainst cadmium intoxication	150 mg/kg diet	[89]
	Seeds	Aqueous extract	Rats	Effect on the kidney in experimental polycystic ovary syndrome female rats	150 mg/kg body weight for 4 weeks	[90]
*Globularia alypum* L.	Aerial parts	Aqueous extract	Rats	Decreases hypertriglyceridemia and ameliorates oxidative status of the muscle, kidney, and heart in rats fed a high-fructose diet	0.5% in diet	[91]
	Whole plant	Chloroform, ethyl acetate and aqueous extracts	Mice	Protective effect against oxonate-induced hyperuricemia and renal dysfunction in mice	100 mg/kg of body weight	[92]
*Glycyrrhiza glabra L.*	Roots	Powder	Rats	Metabolic effects on lipid distribution pattern, liver and renal functions of albino rats	5–10% of Powder in diet	[93]
	Roots	Aqueous extract	Rats	Effect of licorice on adrenal-kidney pituitary axis in rats	100–500 mg/kg of body weight for 15 consecutive days	[94]
*Helianthus annuus* L.	Roots	Petroleum ether extract	Rats	Ameliorative potential on hepatoprotective and some kidney function indices of alloxan induced diabetic rats	100–300 mg/kg of body weight for three weeks	[95]
*Herniaria hirsuta L.*	Aerial parts	Hydro-ethanolic and aqueous extracts	Oxalo-calcic and cystine stones of patients	Dissolution of oxalo-calcic and cystine stones	0.5% of plant extracts in physiological solution (9 g of NaCl /L)	[96]
	Aerial parts	Aqueous extract	Human urine samples	Promoted the precipitation of calcium oxalate particles in urine	0.0625–1 mg/mL	[97]
	Aerial parts	Aqueous extract	Rats	Against calcium oxalate stones induced by ethylene glycol and ammonium chloride	Final concentration was 50 mg/mL (rats received 1 mL/day of extract for 14 days)	[98]
	Aerial parts	Aqueous extract	Rats	Against calcium oxalate urolithiasis risk in rats	The water supply was replaced with an infusion of 4 g/L of plant for 7 days	[99]
	Aerial parts	Aqueous extract	Renal epithelial cells of the Madin Darby canine kidney (MDCK) line	Against adhesion of calcium oxalate monohydrate crystals to cultured renal cells	200 to 800 µg/mL	[100]
	Aerial parts	Aqueous extract	patients	Against cystine stones in different patients with congenital cystinuria	Placing calculations and fragments of calculations cystine in the presence of 20 mL of extract plant for 8 weeks	[101]
*Hordeum vulgare* L.	Seeds	Aqueous and alcoholic seed extracts	Rats	Against ethylene glycol and ammonium chloride-induced urolithasis in rats	200–300 mg/kg of body weight for 35 days	[102]
*Lactuca sativa* L.	Aerial parts	Essential oil	Rabbits	Beneficial effect for the functions and histology of the kidneys	0.1–0.2 mL/kg orally for 17 days	[103]
*Lawsonia inermis* L.	Leaves	Ethanol extract	Rats	Decreased blood glucose level and was able to restore the kidney destruction of alloxan-induced diabetic rats	400–600 mg/kg of body weight for 28 days	[104]
*Lepidium sativum* L.	Seeds	Aqueous extract	Rats	Protective effect against aluminum-induced liver and kidney effects in albino rat	20 mg/kg of body weight for 8 weeks	[105]
	Seeds	Aqueous extract	Rats	Effect on renal glucose reabsorption and urinary TGF-β1 levels in diabetic rats	20 mg/kg of body weight	[106]
*Linum usitatissimum* L.	Seeds	Ethanolic extract	Rats	Renoprotective effect through hemodynamic changes and conservation of antioxidant enzymes in renal ischemia/reperfusion injury in rats	200 mg/kg and 400 mg/kg for 4 weeks	[107]
	Seeds	Aqueous and methanolic extract	Rats	Increased serum estradiol, progesterone, total proteins, total cholesterol, ALT and AST activity, and decreased ovarian cholesterol levels, while it had no effect on kidney function in immature female rats	500 mg/kg daily for 14 days	[108]
	Unspecified	Essential oil	Rats	Ameliorative effects on roundup-induced biochemical and histopathological changes in the liver and kidney of rats	0.5 g/kg of body weight	[109]
*Morus alba* L.	Leaves	Methanol extract	Mice	Antioxidant effect on kidney, testes, spleen and intestine of mice	200–800 mg/kg of body weight for 10 days	[110]
	Leaves	Aqueous extract	Rats	Ameliorative effect against diabetes-induced changes in kidney	1 g/100 g of diet	[111]
	Leaves	Acetone extract	Rats	Ameliorative effect on urine creatinine levels and histology of diabetic rat kidney	90–150 mg/Kg of body weight for 14 days	[112]
	Leaves	Methanol extract	Mice	Ameliorative effect against Schistosoma mansoni-induced renal and testicular injuries in mice	200–800 mg/kg of body weight/day for 10 days	[113]
*Nigella sativa* L.	Whole plant	Essential oil	Rabbits	Against oxytetracycline-induced hepato-renal toxicity in rabbits	2 mL/kg of body weight	[114]
	Seeds	Aqueous and ethanol extracts	Rats	Protective effect on renal ischemia-reperfusion-induced oxidative damage in rats	0.7, 1 and 1.6 g/kg of body weight	[115]
	Seeds	Ethanol extract	Rats	Nephroprotective effect in cisplatin-induced renal injury	50 mg/kg of body weight	[116]
	Seeds	Aqueous extract	Rats	Significantly prevented renal ischemia/reperfusion induced functional and histological injuries	1 g/kg of body weight	[117]
	Seeds	Ethanol extract	Rats	Protective effect against cisplatin-induced renal toxicity and oxidative stress in wistar rats	100–200 mg/kg of body weight for 5 days	[118]
*Ocimum basilicum* L.	Aerial parts	Hydroalcoholic extract	Rats	Against cisplatin models of acute renal failure	100–500 mg/kg of body weight	[119]
	Aerial parts	Essential oils	Rats	Renoprotective effect against diabetes induced renal affection in albino rats	500 mg/kg of body weight/day; given to rats through gastric tube for six weeks)	[120]
	Leaves	Ethanolic extract	Rats	Hepato-renal protective against paracetamol toxicity in rat model	200–400 mg/kg of body weight; once daily for 30 consecutive days)	[121]
	Aerial parts	Hydroalcoholic extract	Rats	Decreased cell injury and apoptosis and preventive effect in kidney tissue damages produced by exposure to electromagnetic field in rats	1.5g/kg of body weight for 40 consequence day	[122]
*Olea europaea* L.	Leaves	Ethanol extract (oleuropein)	Rats	Improvement of blood pressure and cardiac performances, but tends to retain elevated vascular resistance, therefore, reducing the inflow of blood into the brain and kidneys of the spontaneously hypertensive rats	25–50 mg/kg of body weight	[123]
	Leaves	Ethanol extract	Human rhabdmyosarcom cells (RD) (line CCL-136)	Antitumoral activity and the cytotoxicity on renal cells	IC_50_ (75.6 μg/mL)	[124]
	Leaves	Unspecified	Rats	Protective effect against oxidative stress injury generated with renal ischemia reperfusion	100–200 mg/kg of body weight for 15 days	[125]
	Leaves	Ethanol extract	Rats	Up-regulates Nrf2/ARE/HO-1 signaling and attenuates cyclophosphamide-induced oxidative stress, inflammation and apoptosis in rat kidney	100–200 mg/kg of body weight for 15 days	[126]
	Leaves	Essential oil	Rats	Benificial effects on the adrenal-kidney-pituitary axis in rats	100–500 μg/kg of body weight for 14 consecutive days	[127]
*Opuntia ficus-indica* (L.) Mill.	Cladodes	Aqueous extract	Rats	Diuretic effect on rats, and the lyophilized extract has a diuretic and hypotensive effect on normotensive rabbits without deterioration in renal function test	100 mg/kg of body weight	[128]
	Cladodes	Aqueous extract	Rats	Nephroprotective effect on sodium dichromate-induced kidney injury in rats	100 mg/kg of body weight for 40 days	[129]
	Fruits	Prickly pear juice	Rats	Alleviates ethanol-induced kidney injury in rats	20 and 40 mL/kg of body weight	[130]
*Origanum majorana* L.	Whole plant	Essential oil	Rats	Protective effect on hepatic and renal toxicities induced by nickel chloride in male albino rats	0.5 mL/kg of body weight for 4 weeks	[131]
*Petroselinum crispum (Mill.) Fuss*	Fruits	Fresh celery	Women and men urine	Effect on urinary apigenin excretion in human subjects	20 g parsley/10 mL/days	[132]
	Leaves and stems	Ethanolic extract	Rats	Protective effects on ischemia/reperfusion-induced acute kidney injury	100–200 mg/kg of body weight	[133]
	Seeds	Ethanolic extract	Rats	Protective effect on histopathological changes in kidney induced by sodium valproate in male rats	200 mg/kg of body weight/day for 7 weeks	[134]
	Leaves	Parsley juice	Mice	Improving effect against cadmium induces changes in lipid profile, lipid peroxidation and catalase activity in kidneys of male albino mice	0.1 mL of parsley juice/days	[135]
	Leaves	Aqueous extract	Rats	Attenuates serum uric acid level and improves liver and kidney structures in oxonate-induced hyperuricemia rats	3.5–10.5 g/kg of body weight/day	[136]
*Pimpinella anisum* L.	Unspecified	Essential oil	Rats	Decreased the toxicity of aspartame-induced hepatorenal toxicity	0.5 mL/kg of body weight/day; for 2 months	[137]
*Pistacia lentiscus* L.	Fruits	Essential oil	Rabbits	Safe with no adverse effect on liver functions and renal functions with possible anti-glycogenesis activity	1 mL/kg of body weight for 6 consecutive weeks	[138]
*Punica granatum* L.	Fruits	Juice and methanol extract	Rats	Antioxidant properties of pomegranate in hepatic and renal tissues of rats	Juice (3 mL/kg body weight; for 21 days), MtOH (200 mg/kg; body weight; for 21 days)	[139]
	Fruits	Juice	Rats	Reduces lead-induced cell damage in kidney, liver and heart tissue	30–60 μL/days for 5 weeks	[140]
	Seeds	Juice	Rats	Improving effect on diabetes-induced changes in kidney	7.5% of pomegranate seeds in an AIN-76 diet, for a period of two months.	[111]
	Seeds, fruits and peel	Peel MtOH, SOE, fruit juices	Rats	Effects on apoptosis in rat kidney induced by diethylnitrosamine and phenobarbital	Peel MtOH (250 mg/kg; body weight), Fruits juice (250 mg/kg; body weight), SOE (2 mL/kg; body weight)	[141]
	Fruits	Pomegranate juice and methanolic extract of peel	Mice	Improving effect on steroid induced proximal and distal tubular dilatation in mice kidney	Juice (3mL/kg of body weight, for 8 weeks), MtOH peel extract (200 mg/kg of body weight, for 8 weeks)	[142]
	flowers	Hydroalcoholic extract	Rats	Against glycerol-induced acute renal failure in rats	125 and 250 mg/kg of body weight twice daily for 3 days	[143]
*Rosa canina* L.	Fruit	Ethanolic extract	Rats	Protective effects on renal disturbances induced by reperfusion injury in rats	2700 mg/kg of body weight in 3 mL volume through gavage for 7 days	[144]
*Rosmarinus officinalis* L.	unspecified	Tosemary extract containing 40% carnosic acid	rats	Protective effect against etoposide-induced changes in liver and kidney functions, and DNA damage in rats	220 mg/kg of body weight /twice weekly	[145]
	Leaves	Essential oil	Mice	Ameliorants effect on histology and biological parameters of liver and kidney	100–400 mL	[146]
	Leaves	Aqueous extract	Rats	Improving effect on kidney and liver of diabetic rats	0.2 mg/mL/day for 30 days	[147]
*Salvia officinalis* L.	Leaves	Aqueous extract	Mice	Effects on development of mice embryos kidney and some hormonal effect of treated mothers	83.9, 167.8 mg/kg; body weight for 6 weeks	[148] (p. 42)
	Leaves	Essential oil	Mice	Protective effects against hyperlipidemia, liver, and kidney injuries in mice submitted to a high-fat diet	4 mg/kg body weight for 8 weeks	[149]
	Leaves	Ethanol extract	Rats	Preventive effects on chlorpyrifos-and methomyl-induced renal toxicity and oxidative stress in albino rats	50 mg/kg body weight for 4 weeks	[150]
	Leaves	Essential oil	Mice	Protective role against carbon tetrachloride-induced liver and kidney damage in mice	0.1, 0.2, and 0.4 mL/kg body weight for 2 weeks	[151]
*Syzygium aromaticum* (L.) Merr. and L.M.Perry	Clove	Clove oil	Rats	Protective role against acrylamide induced oxidative damage and impairment of liver, kidney, and testicular functions in albino rats	100 and 200 mg/kg of body weight for 21 consecutive days	[152]
*Trigonella foenum-graecum* L.	Seeds	Aqueous extract	Rats	Protective effect on kidney function and morphology in diabetic rats via its antioxidant activity	440–1740 mg/kg of body weight for 6 weeks	[153]
	Seeds	Ethanol extract	Rats	Protective effect against carbon tetrachloride-induced toxicity in liver and kidney of male rat	10% in pellet rat feed for 7 weeks	[154]
	Seeds	Powder	Rats	Against ethylene glycol-induced kidney stone in rats	10 g of fenugreek in 100 mL of water and 10 g in 100 g of standard diet	[155]
	Seeds	Aqueous extract	Rats	Attenuated radiation-induced oxidative stress in liver and kidney tissues	1 g/kg of body weight during 7 days before irradiation	[156]
*Urtica dioica* L.	Leaves	Aqueous extract	Rats	Effects on the expression level of cyclooxygenase-2 and caspase-3 in the liver and kidney of streptozotocin-induced diabetic rats	100 mg/kg of body weight/daily	[157]
	Leaves	Methanolic extract	Rats	Ameliorative effect on acute kidney injury induced by gentamicin in rats	200 mg/kg of body weight/day	[158]
	Leaves	Aqueous extract	Rats	Effects on some blood and urine parameters, and liver and kidney histology in diabetic rats	0.5% infusion of the leaves	[159]
*Vitis vinifera* L.	Grape seeds	Aqueous extract	Mice	Protective role in some biochemical parameters and histological changes in methionine for liver, kidney and heart in mice (Mus musculus)	10–30% mg/kg of body weight during 30 days	[160]
*Zea mays* L.	Stigmata	Aqueous extract	Rats	Antilithiatic effects	The water supply was replaced with an infusion of 2g/L of plant for 7 days	[161]
	Corn silk, leaves	Ethanolic extract	Rats	Improved kidney failure in rat model induced by gentamicin	75 mg/kg of body weight for 4 weeks	[162]
	Corn silk	Aqueous extract	Human urine samples	Solubility of calcium in kidney stones and diuretic effect	2–10% of infuse solution	[163]
*Zingiber officinale* Roscoe	Fresh ginger	Powder	Rats	Protective effect against kidney damage in rats	2.5–5.0% powder of ginger	[164]
	Fresh ginger	Aqueous extract	Mice	Protective effect against injury in the kidney of mice treated with CCL_4_	500 mg/kg of body weight	[165]
	Fresh ginger	Hydro-alcoholic extract	Rats	Effects on treating lead-poisoned kidney of neonatal rats.	2 g/kg of body weight	[166]
	Rhizomes	Ethanol extract	Mice	Protective effect on acute renal failure induced by cisplatin and liver of rats exposed to carbendazim	250 mg/kg of body weight	[167]
	Rhizomes	Powder	Rats	Effects on some physiological parameters and kidney structure in rats	Rats fed with diet contain 5% ZOR Roscoe	[168]
	Rhizomes	Aqueous extract	Rats	Alleviate liver and kidney dysfunctions and oxidative stress induced by mercuric chloride in male rats	125 mg/kg of body weight	[169]
	Rhizomes	Aqueous extract	Rats	Ameliorative effect on the cadmium-induced liver and kidney injury in females’ rats	2 g/L for 40 days	[170]

## Data Availability

All the data are included in the present study.
